# Atlastin-mediated membrane tethering is critical for cargo mobility and exit from the endoplasmic reticulum

**DOI:** 10.1073/pnas.1908409116

**Published:** 2019-06-25

**Authors:** Liling Niu, Tianji Ma, Feng Yang, Bing Yan, Xiao Tang, Haidi Yin, Qian Wu, Yan Huang, Zhong-Ping Yao, Jifeng Wang, Yusong Guo, Junjie Hu

**Affiliations:** ^a^National Laboratory of Biomacromolecules, CAS Center for Excellence in Biomacromolecules, Institute of Biophysics, Chinese Academy of Sciences, 100101 Beijing, China;; ^b^Department of Genetics and Cell Biology, College of Life Sciences, Nankai University, 300071 Tianjin, China;; ^c^Division of Life Science, Hong Kong University of Science and Technology, Hong Kong, China;; ^d^State Key Laboratory of Chemical Biology and Drug Discovery, Department of Applied Biology and Chemical Technology, The Hong Kong Polytechnic University, Hong Kong, China

**Keywords:** endoplasmic reticulum, atlastin, membrane tension, COPII formation, protein mobility

## Abstract

Endoplasmic reticulum (ER) membrane junctions are formed by the dynamin-like GTPase atlastin (ATL). Deletion of ATL results in long unbranched ER tubules in cells, and mutation of human ATL1 is linked to hereditary spastic paraplegia. Here, we demonstrate that COPII formation is drastically decreased in the periphery of ATL-deleted cells. ER export of cargo proteins becomes defective; ER exit site initiation is not affected, but many of the sites fail to recruit COPII subunits. The efficiency of cargo packaging into COPII vesicles is significantly reduced in cells lacking ATLs, or when the ER is transiently fragmented. Cargo is less mobile in the ER in the absence of ATL, but the cargo mobility and COPII formation can be restored by ATL R77A, which is capable of tethering, but not fusing, ER tubules. These findings suggest that the generation of ER junctions by ATL plays a critical role in maintaining the necessary mobility of ER contents to allow efficient packaging of cargo proteins into COPII vesicles.

## Significance

In the early secretory pathway, newly synthesized proteins undergo folding and modifications and then leave the ER through COPII-coated vesicles. How these processes are coordinated and maintained are important but mostly unclear. We show here that ATL, a GTPase that connects ER tubules, controls ER protein mobility and regulates cargo packaging and coat assembly of COPII vesicles. The tethering and fusion activity by ATL likely maintains tension and other necessary parameters for COPII formation in ER membranes. These findings reveal a role of ER shaping in the early secretory pathway and provide insight into behaviors of ER exportation.

In eukaryotic cells, the endoplasmic reticulum (ER) is mainly responsible for protein synthesis, lipid synthesis, and calcium storage (1, 2). Morphologically, as one continuous membrane system, the ER consists of cylindrical tubules and cisternal sheets (1, 3). A characteristic feature of the ER is the reticular network of tubules usually seen in the cell periphery. Tubules, which require high curvature at cross-sections, are shaped by a class of integral membrane proteins, the reticulons and REEPs (4, 5), and subsequently connected in the form of 3-way junctions by dynamin-like GTPase atlastin (ATL) (6, 7).

Deletion or depletion of ATL in mammalian cells causes long unbranched ER tubules (6, 8) indicative of a lack of fusion between tubules. Purified *Drosophila* ATL mediates vesicle fusion in vitro in a GTP-dependent manner (7, 9), providing further evidence of its fusogenic activity. The yeast homolog Sey1p and plant homolog RHD3 have been shown to function similarly (10–12). ATL1 mutations are linked to the human disease hereditary spastic paraplegia (HSP), which is characterized by progressive spasticity and weakness of the lower limbs due to retrograde degeneration of corticospinal axons (13, 14). In *Arabidopsis thaliana*, deletion or mutations of RHD3 are viable but cause prominent defects in root hair cells (12, 15, 16). Thus, ATL-mediated fusion between ER membranes plays an important role in cells with long protrusions. However, ATL proteins are expressed ubiquitously (8), and deletion of RHD3 and either one of its redundant genes leads to lethality in plants (12), suggesting a more fundamental role of ER membrane fusion.

High eukaryotes possess 3 ATLs, with ATL1 predominantly in the nervous system and ATL2/ATL3 in peripheral tissues (8, 17). The enzymatic activity of ATL1 and ATL2 exceeds that of ATL3 (18). ATL is composed of N-terminal cytosolic GTPase, followed by a helical region, 2 transmembrane (TM) segments, and a C-terminal cytosolic tail (CT). Structural and biochemical analyses have revealed that the GTPase domain of ATL forms a dimer upon GTP binding, and GTP hydrolysis causes conformational changes in the associating 3-helix bundle (3HB) (9, 19, 20). The nucleotide-dependent transdimer of ATL tethers apposing membranes, and the swing of 3HB pulls 2 membranes closer for subsequent fusion. In addition, the TM domains of ATL cluster the fusogen in the same membrane and may form intramembrane hairpins (21, 22), and an amphipathic helix in the CT promotes lipid mixing by perturbing the lipid bilayer (21, 23). In vitro studies using purified ATL have demonstrated that continuous GTP hydrolysis is necessary for efficient membrane tethering, and most fusion attempts halt at the tethered state (24). When the purified cytosolic domain of ATL is added to an in vitro assembled tubular ER network with *Xenopus* egg extract, the network is readily disrupted (25), confirming that many junctions are tethered instead of fused. Nevertheless, in cells, the ER membrane system is continuous as long as some 3-way junctions are products of actual fusion.

The generation of the tubular ER network is of great physiological importance. In addition to ATL family-related defects, loss of tubule-forming proteins causes retarded growth in yeast (4) and decreases embryonic survival in *Caenorhabditis elegans* (26), and mutations in Rtn2 and REEP1 are linked to HSP. However, the cellular processes regulated by the tubular ER network are largely unknown. Recent proteomic analysis revealed that 79 proteins are enriched in the ER tubules of yeast cells (27). Functional categorization of these proteins implies that tubular ER may be specialized in lipid synthesis, membrane contacts, and stress signaling. Interestingly, in yeast, COPII vesicles are formed mainly in ER tubules (28), and a set of COPII regulators preferentially localize to the tubular ER (27, 29), suggesting that the tubular ER plays a key role in membrane trafficking.

COPII-coated vesicles deliver cargo proteins from the ER to the Golgi. The assembly and functions of the COPII coat have been studied extensively (30–34). ER-anchored GEF protein Sec12 catalyzes the GTP loading of small GTPase Sar1. GTP-bound Sar1 relocates to the ER and recruits the Sec23/Sec24 heterodimer, forming an inner coat. Sec24 often serves as a cargo adaptor to mediate the packaging of cargo proteins into nascent vesicles, and the growing bud further attracts the Sec13/Sec31 heterodimer, forming the outer coat. COPII is assembled at the ER exit sites (ERESs) marked by scaffolding protein Sec16. However, how the ER morphology interferes with cargo sorting and vesicle formation processes at ERESs remains unclear.

Here, we show that deletion of ATLs causes defects in COPII formation. Lack of ATL results in delayed cargo exit and coat assembly. We also show that COPII defects are likely due to altered cargo mobility and ATL-mediated membrane tethering, but not fusion. These findings provide important insight into the physiological role of the tubular ER network.

## Results

### ATL Regulates COPII Abundance.

**Fig. 1.**
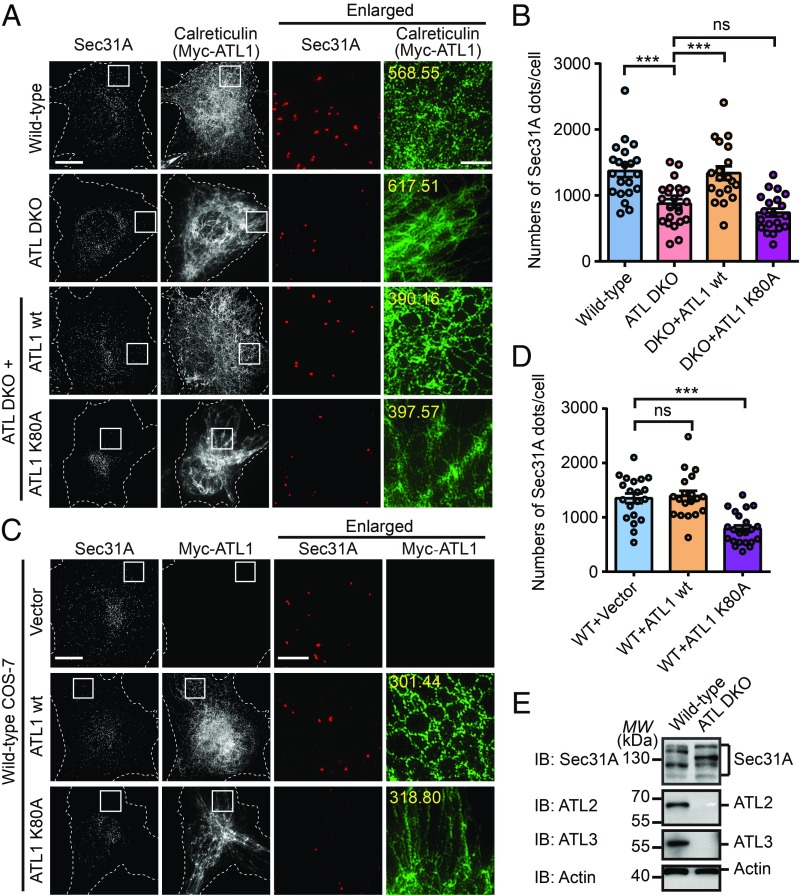
Altered COPII pattern in ATL-deleted cells. (*A*) Nontreated WT, ATL DKO, and Myc-ATL1 WT or Myc-ATL1 K80A transfected ATL DKO COS-7 cells were fixed and stained using anti-Sec31A, anti-calreticulin, or anti-Myc antibodies and imaged using structured illumination microscopy (SIM). Images are projections of 3D datasets (5 μm in *z*). Dashed lines indicate cell boundaries. Yellow numbers indicate the intensities of green fluorescence (ER marker) inside the white square in pixel (Scale bar, 5 μm or 1 μm in enlarged views). (*B*) Quantification of the number of Sec31A-labeled structures in *A* based on SIM (WT, *n* = 21; ATL DKO, *n* = 23; ATL DKO cells transfected with Myc-ATL1 WT, *n* = 19; with Myc-ATL1 K80A, *n* = 20). Data are presented as mean ± SEM. ***P < 0.001 by 2-tailed Student’s *t* test; ns, not significant. (*C*) Wild-type COS-7 cells were transfected with vector, Myc-ATL1 WT or Myc-ATL1 K80A. Twenty-four hours after transfection, cells were fixed and stained using anti-Myc and anti-Sec31A antibodies. SIM images are shown. Yellow numbers indicate the intensities of green fluorescence (ER marker) inside the white square in pixel (Scale bar, 5 μm or 1 μm in enlarged views). (*D*) Quantification of the number of Sec31A-labeled structures in *C* based on SIM (for cells transfected with vector, *n* = 20; with Myc-ATL1 WT, *n* = 19; with Myc-ATL1 K80A, *n* = 22). Data are presented as mean ± SEM. ***P < 0.001 by 2-tailed Student’s *t* test; ns, not significant. (*E*) Cell lysates from wild-type and ATL DKO COS-7 cells were analyzed by immunoblotting using antibodies against Sec31A, ATL2, or ATL3. Actin is used as a loading control. MW, molecular weight (in all figures).

To investigate the role of ATL in membrane trafficking, we used previously generated ATL-deleted COS-7 cells. COS-7 mainly expresses ATL2 and ATL3 (*SI Appendix*, Fig. S1*A*). The 2 ATLs are deleted by the CRISPR/Cas9 system (35), resulting in an ATL double-knockout (DKO) cell line. Two types of frameshift mutations were identified in *ATL2*, both of which cause premature termination of translation. In *ATL3* loci, functional protein production was prevented by homozygous changes (*SI Appendix*, Fig. S1*B*). COPII vesicles were visualized by indirect immunofluorescence using antibodies against a subunit of the outer coat, Sec31A. As expected, Sec31A exhibited a punctate pattern in wild-type COS-7 cells (Fig. 1*A*). These puncta were seen in both the perinuclear region and the cell periphery. However, when the ATLs were deleted, ER tubules became long and unbranched (Fig. 1*A*) and the majority of the Sec31A puncta were concentrated near the nucleus, though cell size remained largely unchanged (Fig. 1*A*). In peripheral areas with equivalent ER marker intensities, the amount of Sec31A puncta was reduced compared with wild type. In addition, the total number of Sec31A puncta in each cell was greatly reduced, as counted by super-resolution fluorescence imaging of the entire cell (Fig. 1*B*), whereas the total ER contents, as judged by the levels of commonly seen ER resident proteins, were not altered (*SI Appendix*, Fig. S1*L*). These results suggest that COPII formation per ER unit is less efficient in ATL DKO cells.

The loss of COPII is likely due to deletion of ATLs, as reintroduction of wild-type ATL1, but not GTP binding-defective K80A mutant (*SI Appendix*, Fig. S1*C*), restored the ER morphology and the abundance and proper distribution of Sec31A-positive puncta (Fig. 1 *A* and *B*). Reduction and redistribution of COPII were also observed when the K80A mutant was overexpressed (*SI Appendix*, Fig. S1*D*) and acted in a dominant-negative manner in wild-type COS-7 cells (Fig. 1*C*). Similarly, depletion of ATL2 and ATL3 in HeLa cells resulted in a decrease in COPII (*SI Appendix*, Fig. S1 *E* and *F*). These results suggest that ATLs play an important role in ER-to-Golgi trafficking.

Next, we tested whether the COPII defects seen in ATL mutant cells are a general consequence of abnormal ER morphology. When GFP-Rtn4a was overexpressed in COS-7 cells, thick bundles of ER tubules were observed as expected (*SI Appendix*, Fig. S2*A*). However, the pattern of Sec31A staining was not affected (*SI Appendix*, Fig. S2*A*). Similarly, when Climp63 was overexpressed to expand ER sheets, Sec31A puncta were still abundant throughout the cell (*SI Appendix*, Fig. S2*A*). In addition, when either Rtn4 or Climp63 was depleted using CRISPR/Cas9 or siRNA (*SI Appendix*, Fig. S2 *D* and *H*), the pattern of Sec31A remained unchanged (*SI Appendix*, Fig. S2 *E* and *I*). These results collectively suggest that the COPII defects seen here are specifically caused by alterations in ATL activity.

As previously reported (8), deletion of ATLs caused some fragmentation of the Golgi apparatus (*SI Appendix*, Fig. S1*H*), particularly the cis-Golgi network (CGN) marked by GM130. However, patterns of β-COP, the coat of COPI, remained similar between wild-type and DKO cells (*SI Appendix*, Fig. S1*I*). In addition, staining of AP1γ1, the γ-subunit of the AP-1 complex localized to the trans-Golgi network (TGN) and endosomes, was not altered in DKO cells compared with wild type (*SI Appendix*, Fig. S1*J*). We also confirmed that the changes seen with Sec31A apply to Sec24C, a subunit of the COPII inner coat, when these coats were costained (*SI Appendix*, Fig. S1*G*). These results suggest that defects caused by ATL deletion are specific to the entire COPII coat.

Next, we tested whether the reduction in COPII puncta is caused by changes in the levels of coat proteins. Compared with wild-type COS-7 cells, no difference in expression was detected when Sar1A, Sec23A, Sec24C, and Sec13 were immunoblotted in DKO cells (*SI Appendix*, Fig. S1*K*). Interestingly, switches in the splicing variants of Sec31A were seen, though the total amount did not change significantly (Fig. 1*E*). Analysis of *Sec31A* transcripts extracted from either wild-type or DKO cells confirmed that the splicing of *Sec31A* was altered upon ATL deletion (*SI Appendix*, Fig. S3*A*), possibly from isoforms 14/18 to 17/9 (*SI Appendix*, Fig. S3 *B* and *C*). Changes in the molecular weight of Sec31A were less likely, due to commonly seen modifications, such as phosphorylation, ubiquitination, or *O*-GlcNAc addition (*SI Appendix*, Fig. S3 *D*–*F*). Notably, the epitopes of Sec31A antibodies used in COPII staining are conserved among isoforms. Taken together, the results rule out the possibility that the COPII abundance defects seen in DKO cells are caused by a shortage of coat proteins. Finally, ER chaperones and key structural proteins were maintained at the same levels between wild-type and DKO cells (*SI Appendix*, Fig. S1*L*), suggesting that a shortage of cargo production is also less likely.

### ATL Mediates ER Export.

**Fig. 2.**
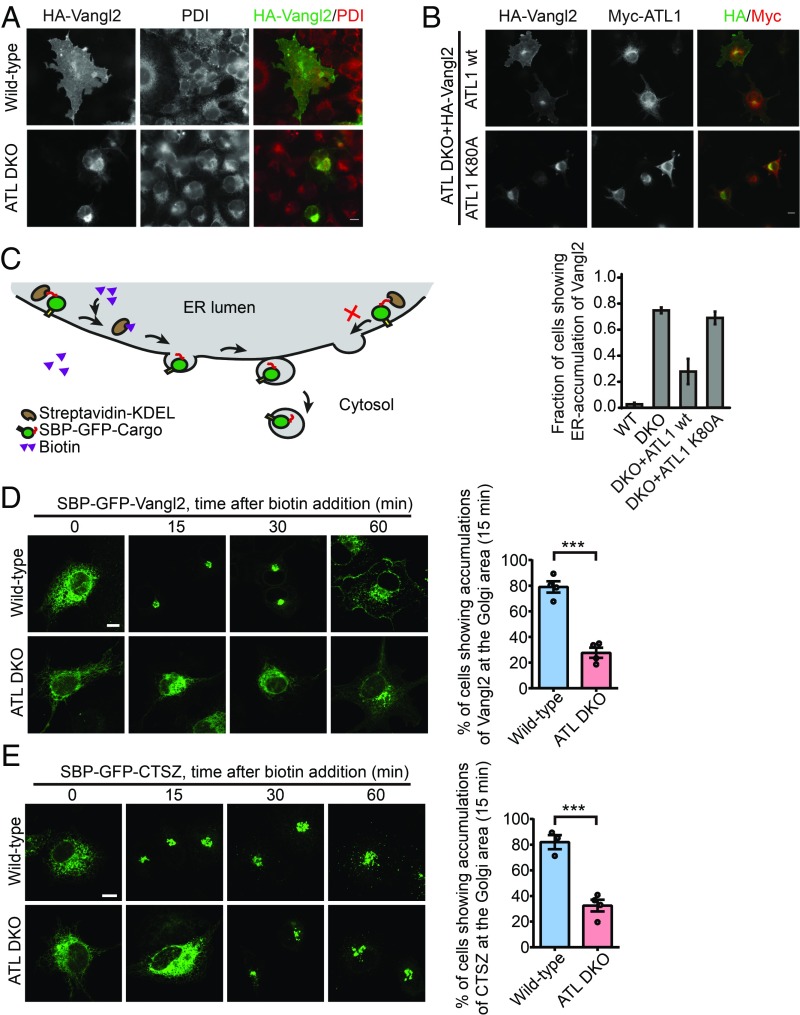
Defective ER export in ATL-deleted cells. (*A*) Wild-type and ATL DKO COS-7 cells were transfected with HA-Vangl2. Twenty-four hours after transfection, cells were fixed and stained using antibodies against HA and PDI. Representative fluorescent images are shown. (*B*) ATL DKO COS-7 cells were cotransfected with HA-Vangl2 and Myc-ATL1, WT or K80A as indicated. Twenty-four hours after transfection, cells were fixed and stained using anti-HA and anti-Myc antibodies. Representative fluorescent images are shown. *Lower* shows quantification of the fraction of cells showing ER accumulation of Vangl2 in the indicated COS-7 cells (*n* = 3, >50 cells were counted for each experiment, data represent mean ± SEM). (*C*) Schematic diagram of the RUSH system. (*D*) Analysis of the trafficking of SBP-GFP-Vangl2 using the RUSH system. Addition of biotin at 0 min released reporters from the ER. Cells were fixed at the indicated time points. Representative confocal images are shown. *Right* bar graphs show the quantified data for the localization of the indicated reporters at 15 min (*n* = 4, each *n* represents a pool of ∼100 transfected cells). Error bars represent mean ± SEM. ***P < 0.001 by 2-tailed Student’s *t* test. (*E*) As in *D*, but with RUSH-CTSZ transfection. *n* = 3 or 4 pools of ∼100 transfected cells). Error bars represent mean ± SEM. ***P < 0.001 by 2-tailed Student’s *t* test (Scale bars, 10 μm).

To test whether ATL activity is important for the ER export process, we analyzed the localization of a planar cell polarity protein, Vangl2. When wild-type COS-7 cells were transfected with plasmids encoding HA-tagged Vangl2, Vangl2 exhibited clear surface localization in 98% of the cells (Fig. 2*A*). In contrast, HA-Vangl2 showed a weak surface-localized pattern and strong accumulation in the perinuclear region, colocalizing with ER marker protein disulfide isomerase (PDI) in 76% of the DKO cells expressing HA-Vangl2 (Fig. 2*A*), suggesting that deletion of ATLs causes an accumulation of Vangl2 at the ER. Coexpression of wild-type Myc-ATL1, but not K80A, significantly reduced the percentage of cells exhibiting ER accumulation of Vangl2 (Fig. 2*B*). Similar results were obtained when ATL2 and ATL3 were depleted in HeLa cells (*SI Appendix*, Fig. S4*A*). Consistent with the COPII abundance analysis, the export of Vangl2 was not influenced by overexpression or depletion of unrelated ER shaping proteins Rtn4a and Climp63 (*SI Appendix*, Fig. S2 *B*, *F*, and *J*).

To monitor cargo exit in a synchronized setting, we established cargo retention using selective hooks (RUSH) (36). Cargo was fused with GFP and streptavidin binding protein (SBP-GFP-Cargo), and initially trapped in the ER by luminal streptavidin fused with ER retention signal KDEL (Fig. 2*C*). Addition of biotin, which competes with streptavidin for SBP, causes a synchronized release of cargo from the ER to downstream compartments. When the integral membrane cargo protein, Vangl2, was tested in the RUSH system in wild-type COS-7 cells, it successfully reached the Golgi within 15 min after biotin addition (Fig. 2*D*). However, the majority of Vangl2 was still trapped in the ER during the same time period in DKO cells (Fig. 2*D*). When SBP-mCherry-Vangl2 and the ER hook protein were transfected into HeLa cells, stable depletion of the 2 major ATLs also caused delayed entrance of Vangl2 into the Golgi (*SI Appendix*, Fig. S4 *B* and *C*). Similarly, soluble cargo cathepsin Z (CTSZ), a lysosomal enzyme, was retained in the ER for a longer time in DKO cells than in wild-type cells when tested by the RUSH system (Fig. 2*E*), even though this soluble cargo is less affected than the membrane-bound cargo Vangl2. Finally, export of SBP-GFP-Vangl2 in the RUSH system was not impacted by overexpression or depletion of Rtn4a and Climp63 (*SI Appendix*, Fig. S2 *C*, *G*, and *K*). Taken together, the results indicate that the loss of ATL delays ER export.

To determine whether various cargo may behave differently, we then tested VSVG, a glycoprotein that serves as a transmembrane model cargo, and the N-terminal signaling domain of Sonic Hedgehog (ShhN), a secretory cargo protein regulating the Sonic Hedgehog signaling pathway (37). Consistent with a previous report (38), VSVG left the ER very quickly (*SI Appendix*, Fig. S4*D*). Differences in VSVG export between wild-type and DKO cells were only seen at earlier time points (less than 15 min after biotin addition). As expected, other reports have demonstrated that VSVG export is not affected in ATL-deleted cells when examined at least 1 h after release (8, 39). In contrast, the export of ShhN was continuously delayed in DKO cells, even 40 min after biotin addition (*SI Appendix*, Fig. S4*E*). A similar delay was seen with another soluble cargo, α1-antitrypsin, at earlier time points (*SI Appendix*, Fig. S4*F*). These results suggest that ATL deletion affects different cargo to different extents.

### ATL Is Critical for Release of Cargo Proteins into COPII Vesicles.

**Fig. 3.**
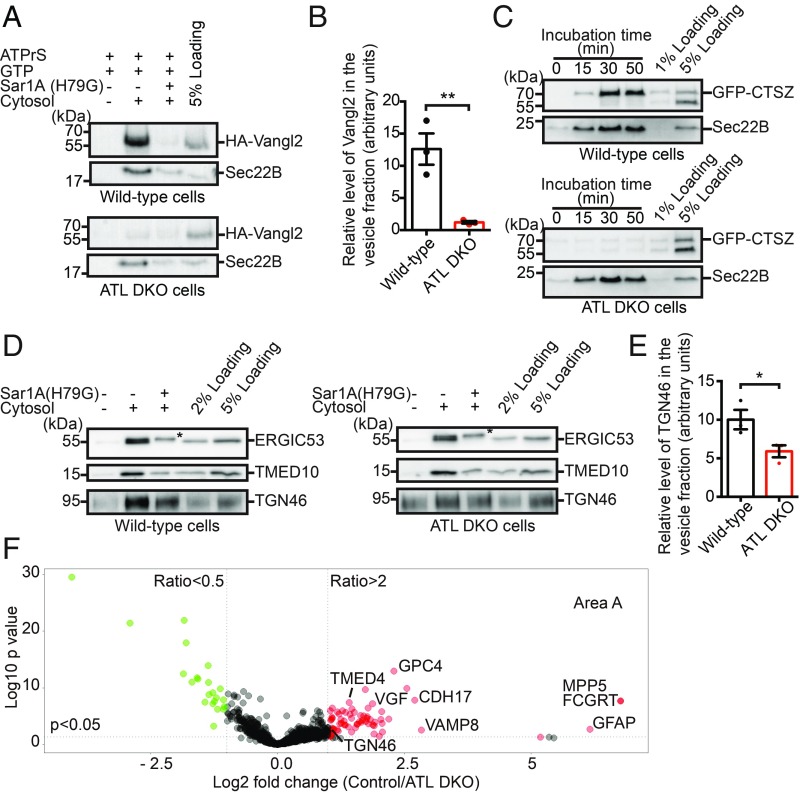
Reduced COPII vesicle packaging in ATL-deleted cells. (*A*) Wild-type and ATL DKO COS-7 cells were transfected with HA-Vangl2. Twenty-four hours after transfection, cells were incubated at 15 °C for 2 h and the vesicle release reaction was performed by incubating the digitonin-permeabilized cells with the indicated reagents. Vesicle fractions were then isolated and analyzed by immunoblotting using antibodies against HA or Sec22B. (*B*) Quantification of the levels of HA-Vangl2 in the vesicle fraction normalized to 5% loading (*n* = 3, mean ± SEM **P < 0.01 by 2-tailed Student’s *t* test). (*C*) Wild-type COS-7 and ATL DKO COS-7 cells were transfected with GFP-CTSZ. Twenty-four hours after transfection, the vesicle release reaction was performed using the indicated reagents. Vesicle fractions from different time points were then analyzed by immunoblotting using antibodies against GFP or Sec22B. (*D*) The vesicle release reaction was performed using wild-type and ATL DKO COS-7 cells. Vesicle fractions were then isolated and analyzed by immunoblotting using the indicated antibodies. Asterisks indicate the nonspecific signal detected by the antibodies. (*E*) Quantification of the levels of TGN46 in the vesicle fraction normalized to the 5% loading (*n* = 3, mean ± SEM; *P < 0.05 by 2-tailed Student’s *t* test). (*F*) The vesicle release reaction was performed using wild-type and ATL DKO COS-7 cells in the presence of rat liver cytosol. Vesicle fractions were then isolated and resuspended in RapiGest SF surfactant. The proteins in the vesicle fractions were trypsin digested and analyzed by label-free mass spectrometry. The mean log2 fold changes in the identified proteins in the control group over the ATL DKO group were calculated and plotted against the minus log10 *P* value. ATPrS: ATP regeneration system. Data include 3 biological repeats.

To test whether ER accumulation of cargo in DKO cells was caused by defective ER export or by enhanced ER retrieval, we performed an in vitro assay that reconstitutes vesicular release of cargo from the ER (40). As Vangl2 did not exhibit apparent ER localization in wild-type COS-7 cells (Fig. 2*A*), likely due to constitutive and quick export in the steady state, we performed the vesicle release assay after incubating cells at 15 °C to accumulate newly synthesized Vangl2 at the ER. HA-Vangl2–expressing cells were permeabilized by digitonin and incubated with rat liver cytosol, GTP, and an ATP regeneration system. After incubation, the released vesicles were separated from the donor membranes by centrifugation and analyzed by immunoblotting. Sec22B, a v-SNARE that directly binds to COPII and regulates ER-to-Golgi trafficking, was readily released from the ER when all components were added (Fig. 3*A*). The efficiency of release was significantly reduced when a GTPase-defective mutant of Sar1A (H79G), which acts in a dominant-negative manner, was included (Fig. 3*A*). Similarly, we reproducibly detected that vesicular release of Vangl2 was enhanced by cytosol (Fig. 3*A*) and abolished by Sar1A (H79G) (Fig. 3*A*). In contrast, we did not detect clear vesicular release of Vangl2 in DKO cells (Fig. 3 *A* and *B*). The levels of Sec22B in the vesicle fraction were similar in the wild-type and DKO cells (Fig. 3*A*), suggesting that packaging of Sec22B into COPII vesicles was not interrupted by ATL deletion. These results indicate that ATL deletion causes defects in the packaging of cargo, in this case Vangl2, but not necessarily components, such as Sec22B, into COPII vesicles.

We then tested the budding of CTSZ-containing vesicles. In wild-type cells, efficient release of GFP-CTSZ was readily seen after 30 min of incubation. However, very little release of GFP-CTSZ was achieved with DKO cells, even after 50 min (Fig. 3*C*), but release of Sec22B was not changed. Similarly, Sar1A (H79G) reduced the efficiency of release of both Sec22B and CTSZ into vesicles (*SI Appendix*, Fig. S5*A*). We also reconstituted vesicular release of HA-tagged ShhN from semiintact cells. ShhN showed a cytosol-dependent release into transport vesicles that was partially inhibited by Sar1A (H79G) (*SI Appendix*, Fig. S5*B*). Like other cargo, the efficiency of release of ShhN into transport vesicles was significantly reduced in ATL DKO cells compared with wild-type cells (*SI Appendix*, Fig. S5*B*).

ATL deletion did not affect the release of endogenous Sec22B into transport vesicles. Therefore, we analyzed whether it interferes with other endogenous cargo proteins, including ERGIC53, TMED10, and TGN46. Sec22B and ERGIC53 cycle between the ER and Golgi, and they directly interact with the COPII coat (41–43). TMED10 is a member of the p24 family predicted to be cargo receptors in the early secretory pathway and to bind to COPII (44). TGN46 is a trans-Golgi–localized cargo protein (45). To accumulate newly synthesized endogenous cargo proteins in the ER, we preincubated cells at 15 °C before applying cells to the vesicle formation assay. We found that deletion of ATLs did not affect the release of ERGIC53 and TMED10 into transport vesicles (Fig. 3*D*). In contrast, the efficiency of release of TGN46 was significantly reduced in ATL DKO cells (Fig. 3 *D* and *E*).

Ribophorin 1, an ER resident protein, had a cytosol-dependent and Sar1A (H79G)-insensitive signal in the vesicle fraction, albeit with low efficiency (*SI Appendix*, Fig. S5*C*). However, a similar band was detected in rat liver cytosol alone (*SI Appendix*, Fig. S5*D*). Thus, the low-level release of Ribophorin 1 in the vesicle fraction is likely contamination by the rat liver cytosol used in the assay. Another ER resident protein, calreticulin, was not detected in the vesicle fraction when the vesicle budding reaction was performed, utilizing either wild-type COS-7 cells or ATL DKO COS-7 cells (*SI Appendix*, Fig. S5*E*). These results suggest that ER resident proteins cannot be efficiently packaged into transport vesicles in the in vitro system.

To systematically identify endogenous cargo proteins with reduced packaging into transport vesicles in ATL DKO cells, we performed large-scale quantitative proteomics on vesicles collected from wild-type control and ATL DKO COS-7 cells. The proteins in the buoyant membrane fractions were first analyzed by SDS/PAGE and Coomassie Blue staining. A series of protein bands was recovered in the buoyant membrane fraction when the assay was performed in the presence of cytosol (*SI Appendix*, Fig. S5*F*, lanes 2 and 4). Several protein bands had higher staining intensities in the control group than in the DKO group (*SI Appendix*, Fig. S5*F*, marked by asterisks), indicating candidates for affected cargo. Consistent with small-scale tests, the levels of Sec22B and ERGIC53 in the buoyant membrane fraction were similar in both the control and DKO groups (*SI Appendix*, Fig. S5*G*, lanes 2 and 5).

Next, we performed a label-free quantitative mass spectrometry analysis to compare protein profiling of the vesicle fractions in the control vs. DKO groups based on 3 biological repeats. A total of 913 proteins were identified with 2 or more unique peptides (*SI Appendix*, Table S1, sheet 1). The fold changes in the identified proteins in each control group compared with the DKO group were quantified. These changes were then normalized according to the average fold change in Sec22B and ERGIC53, which was set to 1. Based on the fold changes, a *P* value was calculated and plotted against the mean log2 fold changes (Fig. 3*F*). Through this quantification approach, 71 proteins, including TGN46, were identified as having more than 2-fold enrichment in the control group over the DKO group (*P* < 0.05; Fig. 3*F*, area A). Forty-five (∼63%) were cargo proteins; 39 were predicted by UniProt to be non-ER resident transmembrane proteins that need exportation from the ER, and 6 as soluble or GPI-anchored proteins (*SI Appendix*, Table S1, sheet 2). Twenty-two proteins were identified with more than 2-fold enrichment in the DKO group over the control group (*P* < 0.05; *SI Appendix*, Table S1, sheet 3). We identified 8 unique peptides that match ATL3 in the vesicle fraction produced by wild-type COS-7 cells. Unfortunately, one of the peptides was also detected in the vesicle fraction generated by the DKO cells. A possible explanation is that rat liver cytosol may contain a small amount of ATL3 that associated with vesicles after the vesicle formation assay. Taken together, these results confirm that deletion of ATL causes defects in the packaging of a variety of endogenous cargo proteins, mostly transmembrane proteins, into transport vesicles.

Trapped cargo causes ER stress (46–48). Therefore, we tested whether ATL deletion or depletion triggers the unfolded protein response (UPR) by delaying cargo export. One of the hallmarks of UPR activation is PERK-mediated phosphorylation of eIF2α. When tested in DKO cells, we noticed a consistent increase in eIF2α phosphorylation (*SI Appendix*, Fig. S6), though the degree of increase was less than that induced by thapsigargin (TG) treatment. Similar signs of UPR were detected when ATLs were depleted in COS-7 or HeLa cells (*SI Appendix*, Fig. S6). These results confirm that ATL activity is required for ER homeostasis, which is likely coupled to ER export.

### ATL Activity Regulates Recruitment of COPII Coats at the ERES and Affects ER Protein Mobility.

**Fig. 4.**
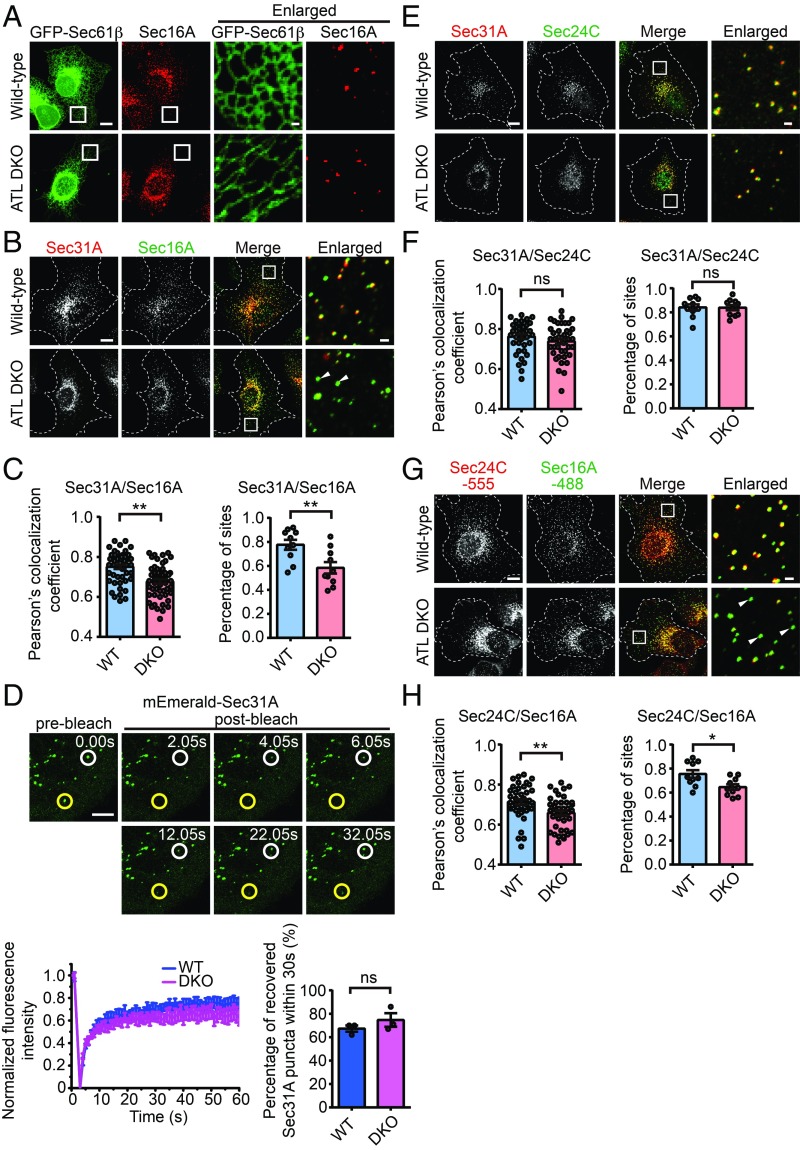
Uncoupling of Sec16A and Sec31A in ATL-deleted cells. (*A*) Wild-type and ATL DKO COS-7 cells transfected with GFP-Sec61β were immunostained for endogenous Sec16A. Representative confocal images are shown (Scale bars, 10 μm or 1 μm for enlarged views). (*B*) Wild-type and ATL DKO COS-7 cells were fixed and immunostained with anti-Sec16A and anti-Sec31A antibodies. Representative confocal images are shown (Scale bars, 10 μm or 1 μm for enlarged views). Dashed lines indicate cell boundaries. (*C*, *Left*) Quantification of Pearson’s colocalization coefficient between Sec16A and Sec31A in a 100-μm^2^ region away from the nucleus (wild type, *n* = 50; ATL DKO, *n* = 52). (*C*, *Right*) Quantification of the proportion of Sec31A-labeled structures colocalized with Sec16A-labeled structures (*n* = 10 pools of ∼100 Sec16A-labeled structures). Date are presented as mean ± SEM. **P < 0.01 by 2-tailed Student’s *t* test. (*D*) Wild-type and ATL DKO COS-7 cells were transfected with plasmids encoding mEmerald-Sec31A. Twenty-four hours after transfection, Sec31A turnover was analyzed by FRAP. Each region indicated by white and yellow circles (*Upper*) was photobleached and fluorescence recovery followed over time (Scale bar, 5 μm). Recovery curves (wild type, *n* = 9; ATL DKO, *n* = 11) and quantification of the proportion of mEmerald-Sec31A puncta recovered within 30 s (*n* = 3 pools of ∼80 bleached region) are shown. (*E*) As in *B*, but with anti-Sec24C and anti-Sec31A antibody staining. (*F*, *Left*) Quantification of Pearson’s colocalization coefficient between Sec24C and Sec31A in a 100-μm^2^ region away from the nucleus (*n* = 40). (*F*, *Right*) Quantification of the proportion of Sec31A-labeled structures colocalized with Sec24C-labeled structures (*n* = 10 pools of ∼100 Sec24C-labeled structures). Data are presented as mean ± SEM; ns, not significant. (*G*) As in *B*, but with anti-Sec24C and anti-Sec16A antibody staining. (*H*, *Left*) Quantification of Pearson’s colocalization coefficient between Sec24C and Sec16A in a 100-μm^2^ region away from the nucleus (*n* = 40). (*H*, *Right*) Quantification of the proportion of Sec31A-labeled structures colocalized with Sec24C-labeled structures (*n* = 10 pools of ∼100 Sec24C-labeled structures). Data are presented as mean ± SEM. *P < 0.05, **P < 0.01 by 2-tailed Student’s *t* test.

To dissect the specific defective steps in COPII assembly, we checked the initiation of COPII formation by staining Sec16A, a commonly used marker and scaffold of ERESs. Similar to Sec31A, and as previously seen, Sec16A formed puncta throughout the cell (Fig. 4*A*). This distribution pattern remained largely unchanged in DKO cells (Fig. 4*A*). When Sec31A and Sec16A were costained, the majority of their puncta overlapped in wild-type cells but partially segregated in DKO cells (Fig. 4 *B* and *C*), suggesting a defective COPII coat occupancy at ERESs. Interestingly, turnover of COPII subunit, measured by fluorescent recovery after photobleaching (FRAP) assay of mEmerald-Sec31A, was very similar in wild-type and DKO cells (Fig. 4*D*), even though 2 types of Sec31A puncta were found: one actively remodeling and the other very steady. We confirmed that outer coat assembly is tightly coupled to inner coat assembly. Staining of endogenous Sec24C, a component of the inner COPII coat, superimposed nicely with Sec31A (Fig. 4 *E* and *F*), but segregated largely with Sec16A (Fig. 4 *G* and *H*). We also confirmed that an isoform switch of Sec31A in DKO cells did not affect the interactions with Sec16A (*SI Appendix*, Fig. S7). These results indicate that, in ATL-deleted cells, ERESs are efficiently formed but COPII coat assembly often fails to follow at these sites.

**Fig. 5.**
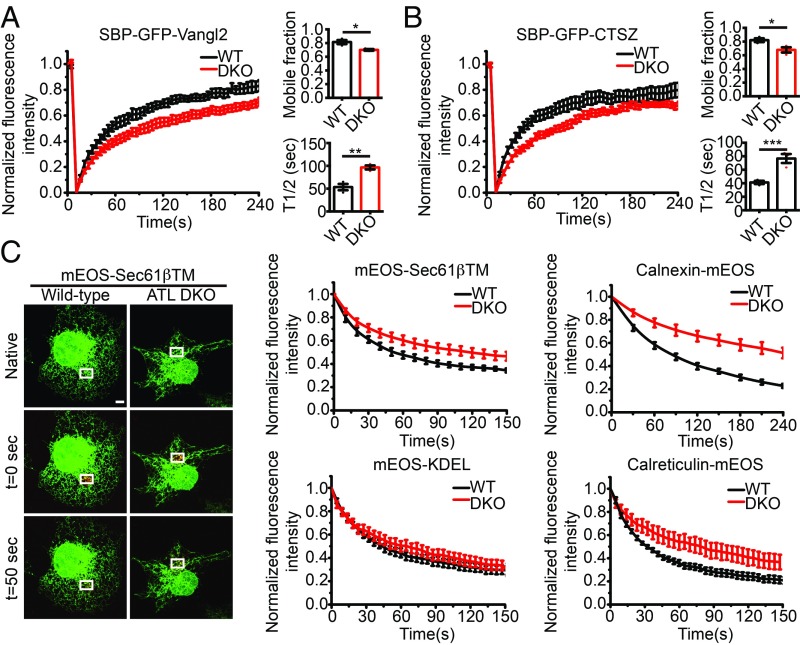
Reduced protein mobility in the ER of ATL-deleted cells. (*A* and *B*) FRAP in the ER of wild-type and ATL DKO cells expressing RUSH cargo proteins. Cells were transfected with SBP-GFP-Vangl2 in *A* or SBP-GFP-CTSZ in *B* for 18 h. FRAP was performed immediately after biotin addition. Recovery curves (mean ± SEM), mobile fractions, and average recovery halftime (T1/2) were derived from FRAP experiments. *n* = 3, ≥ 14 cells per group; bars are presented as mean ± SEM; *P < 0.05, **P < 0.01, ***P < 0.001 by 2-tailed Student’s *t* test. (*C*) FLAP in the ER of wild-type and ATL DKO cells expressing ER-targeted photoswitchable (green to red) fluorescent reporters. ER membrane protein reporters include mEOS-Sec61βTM and calnexin-mEOS. ER luminal protein reporters include calreticulin-mEOS and mEOS-KDEL. Taking mEOS-Sec61βTM as an example, reporter was photoswitched in a small area (white rectangle) next to the nucleus, after which the switched fluorophores diffused to other regions of the ER. The frame just before photoswitching is indicated as native, and the frame immediately following photoswitching as t = 0 s. The fluorescence loss over time was measured for the photoswitched region. The initial fluorescence intensity was set to 1. The curves show the mean fluorescence intensity ± SEM. *n* > 3, ≥ 16 cells per group (Scale bar, 5 μm).

Recently, ATL depletion was reported to cause delayed targeting of inner nuclear membrane proteins synthesized in the peripheral ER (38). Therefore, we tested whether ATL deletion impacts protein mobility in the ER in general. FRAP was performed after the RUSH cargo, and SBP-GFP-Vangl2 was released from the ER by biotin. Areas with equivalent ER signals in wild-type and DKO cells were selected and bleached. The mobility of the integral membrane cargo was clearly reduced in DKO cells (Fig. 5*A*). A similar reduction in mobility was observed with soluble cargo SBP-GFP-CTSZ (Fig. 5*B*) and SBP-GFP-α1-antitrypsin (*SI Appendix*, Fig. S8*A*). Notably, the mobility of SBP-GFP-CTSZ before biotin addition was very similar to the mobility after biotin addition (*SI Appendix*, Fig. S8*B*).

Next, we measured the mobility of other ER-localized proteins using a different approach. Protein fused with an mEOS fluorescent protein (49), which is photoswitchable, was transfected into wild-type or DKO cells. When areas of given amounts of ER were laser treated to induce a green-to-red switch of mEOS, the loss of red signal from the treated area was used to indicate mobility of the tagged protein. We termed this assay fluorescent loss after photoswitching (FLAP). When the TM domain of Sec61β was tagged with mEOS and measured by FLAP, it moved slower in DKO cells (Fig. 5*C*). When calnexin-mEOS, another ER resident integral membrane protein, was measured, its mobility was further reduced in DKO cells. The mobility of ER luminal proteins mEOS-KDEL and calreticulin-mEOS was also slowed in DKO cells, though to a lesser extent than membrane proteins (Fig. 5*C*). We also compared FLAP with FRAP by testing calnexin-mEOS and calreticulin-mEOS with FRAP, and obtained the same results (*SI Appendix*, Fig. S8*C*). Consistent with the above analysis, overexpression of neither Rtn4a nor Climp63 altered the mobility of ER proteins, including calnexin and calreticulin (*SI Appendix*, Fig. S8*D*). Collectively, these results suggest that deletion of ATL reduces cargo mobility.

**Fig. 6.**
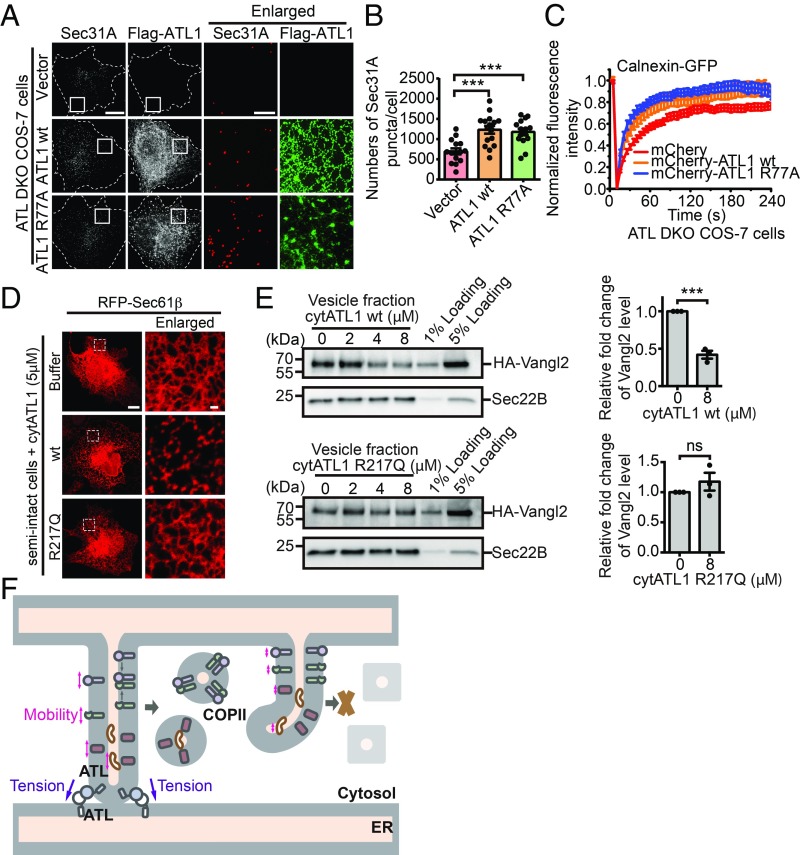
The role of ATL-mediated membrane tethering in COPII formation. (*A*) ATL DKO COS-7 cells were transfected with vector, Flag-ATL1 WT, or Flag-ATL1 R77A. Twenty-four hours after transfection, cells were fixed and stained using anti-Flag and anti-Sec31A antibodies. Representative SIM images are shown (Scale bars, 5 μm or 1 μm for enlarged views). (*B*) Based on SIM images, the number of Sec31A-labeled structures in ATL DKO COS-7 cells transfected with empty vector (*n* = 15), Flag-ATL1 WT (*n* = 16), or Flag-ATL1 R77A (*n* = 15) was quantified. Data are presented as mean ± SEM. ***P < 0.001 by 2-tailed Student’s *t* test. (*C*) FRAP in the ER of ATL DKO cells transfected with indicated plasmids (vector, *n* = 8; mCherry-ATL1 WT, *n* = 8; Flag-ATL1 R77A, *n* = 11). (*D*) Wild-type COS-7 cells transfected with RFP-Sec61β were semipermeabilized by digitonin and preincubated with buffer or 5 μM purified His-cytATL1 WT or His-cytATL1 R217Q for 20 min at 37 °C. Cells were then imaged using confocal microscopy. Representative images from 3 individual experiments are shown (Scale bars, 10 μm or 1 μm for enlarged views). (*E*) Wild-type COS-7 cells were transfected with HA-Vangl2. Twenty-four hours after transfection, the vesicle release reaction was performed in the presence of purified His-cytATL1 WT or His-cytATL1 R217Q. Vesicle fractions were then analyzed by immunoblotting. Quantification of the levels of HA-Vangl2 in the vesicle fraction is normalized to the 5% loading (*n* = 3, mean ± SEM). ***P < 0.001 by 2-tailed Student’s *t* test; ns, not significant. Data are representative of 3 biological repeats. (*F*) A model for the role of ATL in COPII formation. See *Discussion* for details.

Membrane tension has been reported to regulate the lateral mobility of embedded proteins (50). Therefore, we hypothesized that ATL activity is critical for maintaining the necessary tension and subsequent movements of ER proteins. Tension in ER tubules can be achieved by either tethering or fusion between tubules. To this end, we introduced ATL1 R77A mutant into DKO cells. R77 in ATL1 is thought to catalyze GTP hydrolysis; its substitution with alanine retains nucleotide binding and dimerization of ATL1 but abolishes fusion activity. In R77A-expressing DKO cells, the tubular ER network appeared to be partially restored (*SI Appendix*, Fig. S9*A*). As seen previously (11), R77A was enriched at 3-way junctions (Fig. 6*A* and *SI Appendix*, Fig. S9*A*). The amount of junctions in these cells, likely mediated by membrane tethering, but not fusion, was significantly increased compared with nontransfected DKO cells (*SI Appendix*, Fig. S9*B*). Consistent with our hypothesis, R77A rescued the COPII amounts indicated by Sec31A (Fig. 6 *A* and *B*) and protein mobility demonstrated by calnexin (Fig. 6*C*). These results indicate that ATL-mediated membrane tethering is important for cargo mobility and COPII assembly at the ER.

We also performed the vesicle budding assay in the absence or presence of purified ATL cytosolic domain (cytATL1 WT) (*SI Appendix*, Fig. S9 *C* and *D*). CytATL1 interacts with the cytosolic domain of endogenous ATL, which prevents dimerization of endogenous ATL, disrupts ATL-mediated membrane tethering, and subsequently causes ER fragmentation both in vitro and in cells (25, 51). The addition of wild-type cytATL1 to the digitonin-permeabilized cells fragmented the ER network. In contrast, the ER network was not disrupted by a dimerization-deficient mutant of ATL1 (cytATL1 R217Q) (Fig. 6*D*). The efficiency of packaging Vangl2 in transport vesicles was inhibited by wild-type cytATL1 in a concentration-dependent manner, whereas cytATL1 R217Q had marginal effects (Fig. 6*E*). These results indicate that disruption of ATL-mediated membrane tethering, which reduces membrane tension, inhibits the efficiency of packaging cargo protein into COPII vesicles.

## Discussion

Our previous work demonstrated that ATL and its homologs mediate fusion of the ER, particularly the tubular network, in a GTP-dependent manner (6, 9, 21, 24, 52, 53). However, probing the direct impact on ATL mutations or deletion is difficult, as the resulting ER morphological defects are profound and dramatic. Here, we show that ATL deletion affects COPII formation and cargo exit from the ER. The defects are likely caused by altered membrane tension, which in turn decreases the mobility of ER proteins, including the export of cargo.

The lateral tension of ER membranes can be maintained by pulling the tip of a tubule via microtubule-dependent mechanisms or by holding it onto an adjacent tubule, which leads to the formation of a 3-way junction. ATL plays an essential role in the latter case. As expected, ATL deletion drastically reduces the number of junctions in the ER, very likely reducing membrane tension. Even when the network is transiently disrupted by the addition of purified cytATL1, a similar impact can be achieved. Interestingly, an ATL mutant, R77A, which is tethering competent but fusion deficient, is able to rescue lateral mobility defects and COPII formation. This evidence indicates that tethered ER junctions are sufficient to maintain membrane tension and allow efficient cargo export. Unfortunately, the majority of ATL1 mutations linked to HSP fail to even tether membranes (9). Notably, tension mediated by microtubule interaction and ATL activity, even though they are likely of very different degrees, may ultimately be coordinated, as microtubule-stabilizing reagent taxol has been shown to partially rescue defects in ATL-mutated cells (54). However, altered membrane tension may not explain all defects seen in ATL deletion. ER exportation is drastically affected when ATL is completely missing (Fig. 3) but only partially compromised when endogenous ATL is antagonized by purified cytATL (Fig. 6). In the latter case, tension is disrupted, as judged by ER fragmentation, but some ER exportation capacity is present. Presumably, ATL may also act in a tension-independent manner.

ER luminal particles have been shown to be propelled in an active flow driven by tubule contraction events (55). The force that generates tubule contraction can be regulated by membrane tension. Lateral membrane tension would modulate spaces between lipid molecules, directly influencing the mobility of membrane-embedded proteins. In experiments using single particle tracking in reconstituted giant unilamellar vesicles, integral membrane proteins with a curved shape moved faster laterally when membrane tension was increased (50). It is possible that the same principle could be applied to noncurved proteins in curved membranes, such as ER tubules. We have observed that mobility changes are more prominent with integral membrane proteins than luminal proteins. How soluble proteins inside the ER are affected by ATL activity remains to be investigated. One possible scenario is that tension-containing ER membranes have a high frequency of membrane fluctuation, facilitating active luminal flow (55). Alternatively, ATL activity may be linked to ER vibration (56), which subsequently affects the mobility of luminal contents.

We hypothesize that ATL-mediated membrane tethering provides force to maintain membrane tension, thereby facilitating the mobility of cargo proteins at the ER (Fig. 6*F*). Increasing the mobility of proteins at the ER may increase the chance of cargo proteins meeting with their cargo receptors to be efficiently packaged into transport vesicles. Increasing protein mobility may also facilitate cargo proteins meeting with molecular chaperones or modification enzymes in the ER lumen to help them be folded or modified correctly. This process allows the cargo proteins to escape from the clutches of the ER quality control system (41). Interestingly, disrupting ATL-mediated membrane tethering did not affect the packaging of Sec22B, ERGIC53, and TMED10 into COPII vesicles. Both Sec22B and ERGIC53 directly bind COPII and constitutively traffic between the ER and Golgi. TMED10 belongs to the p24 family, which is also predicted to directly bind COPII and cycle between the ER and Golgi. The ER exit sites are shown to be juxtaposed to the ER arrival sites for COPI vesicles (57). We hypothesize that these cargo proteins do not need to be associated with cargo receptors to be sorted into COPII vesicles and are readily packaged into COPII vesicles after they are retrieved back to the ER; thus, their ER export processes are not interrupted by deleting ATL.

Previous studies using a *Drosophila* model have shown that disruption of *atl* in motor neurons causes defects in locomotion and impairs presynaptic function (58, 59). Similarly, depletion of *atl* in zebrafish causes a severe decrease in larval mobility, presumably due to defective trafficking of BMP receptor (60). These findings are consistent with ATL activity playing a direct role in membrane trafficking. It is also possible that, by regulating ER morphology, ATL indirectly influences calcium signaling (61), endosomal movements (62), and microtubule dynamics (63).

## Materials and Methods

### Cloning and Plasmids.

The generation of Myc-ATL1 (8), Myc-ATL1 K80A (8), Flag-ATL1 (52), His-cytATL1 (9), HA-Vangl2 (40), GFP-Rtn4a (3), and GFP-Sec61β (64) were described previously. All plasmids were confirmed by sequencing.

### Mammalian Cell Culture, Transfection, and Immunofluorescence.

COS-7 cells (ATCC) were maintained in DMEM (Corning) supplemented with 10% FBS (Gibco) at 37 °C in 5% CO_2_.

Transfections were performed using Lipofectamine 3000 or Lipofectamine 2000 (Invitrogen) according to the manufacturer’s instructions. Confocal and super-resolution imaging was performed as described previously (65).

### RUSH Transport Assay.

COS-7 cells were cultured as described above and transfected using calcium phosphate (Promega) according to the manufacturer’s instructions. Release of the RUSH reporters was induced by the addition of 40 μM biotin (Sigma) in the presence of 100 μg/mL cycloheximide (Merck-Millipore) as described previously (36). Images were acquired by Zeiss LSM700 confocal microscopy.

### FRAP and FLAP Assays.

COS-7 cells were cultured and transfected as described above. FRAP experiments were performed using a Zeiss LSM880 confocal microscope (PeCon GmbH, Erbach, Germany) and processed with Zen software. FLAP images were obtained using an Olympus FV1200 laser scanning confocal microscope (Olympus, Tokyo, Japan) and analyzed in FV10-ASW2.0 and OriginPro8.

### Vesicular Release Assay.

The vesicular release assay was described previously (40).

### Statistical Analysis.

Averages and SEMs from at least 3 independent experiments are shown in figures when applicable. Sample sizes were chosen without performing statistical tests, but are based on studies with similar experimental designs and on the known variability of the assay. The data are presented as the mean ± SEM. Significance was determined by a Student’s *t* test. All *P* values <0.05 were considered significant. Calculations were performed using GraphPad Prism 6 software.

Further details on methods are provided in *SI Appendix*, *Materials and Methods*.

## Supplementary Material

Supplementary File

Supplementary File
