# Supplementary material for: Atlastin-mediated membrane tethering is critical for cargo mobility and exit from the endoplasmic reticulum
Source: Proc Natl Acad Sci U S A. 2019 Jun 25;116(28):14029–38. doi: 10.1073/pnas.1908409116 (PMC6628656; doi:10.1073/pnas.1908409116)
Supplement: Supplementary File [file pnas.1908409116.sapp.pdf]

Supplemental Information (SI) Appendix

Atlastin mediated membrane tethering is critical for cargo mobility and exit from the endoplasmic reticulum

Liling Niu[1,2,5], Tianji Ma[3,5], Feng Yang[3], Bing Yan[1], Xiao Tang[3], Haidi Yin[4], Qian Wu[4], Yan Huang[3], Zhong-Ping Yao[4], Jifeng Wang[1], Yusong Guo[3,*], Junjie Hu[1,*]

[1]National Laboratory of Biomacromolecules, CAS Center for Excellence in Biomacromolecules, Institute of Biophysics, Chinese Academy of Sciences, Beijing 100101, China

[2]Department of Genetics and Cell Biology, College of Life Sciences, Nankai University, Tianjin 300071, China

[3]Division of Life Science, Hong Kong University of Science and Technology, Hong Kong, China

[4]State Key Laboratory of Chemical Biology and Drug Discovery, Department of Applied Biology and Chemical Technology, The Hong Kong Polytechnic University, Hong Kong, China

[5]These authors contributed equally

[*]To whom correspondence should be addressed: huj@ibp.ac.cn (J.H.)

guoyusong@ust.hk (Y.G.)

Includes:

SI materials and methods

SI figures, Fig S1-S9

SI MATERIALS AND METHODS

Cloning and plasmids

The generation of Myc-ATL1 (1), Myc-ATL1 K80A (1), Flag-ATL1 (2), His-cytATL1 (3), HA-Vangl2 (4, 5), GFP-Rtn4a (6), and GFP-Sec61β (7) were described previously.

The constructs encoding His-cytATL1 R217Q and Flag-ATL1 R77A were generated using the QuikChange Site-Directed Mutagenesis Kit (Stratagene). GFP-CTSZ was PCR-amplified from a cDNA library in HEK293T cells. The GFP tag was inserted downstream from the signal sequence and cloned into pcDNA4/TO vector (Invitrogen). The constructs encoding HA-tagged Sec31A were generated by cloning different isoforms of Sec31A, followed by the 2xHA tag in pCI-neo vector (Promega). HA-Climp63 was PCR-amplified from mouse cDNA using primers containing an N-terminal HA tag and inserted into pcDNA5/TO (Invitrogen). mCherry-Rtn4a and mCherry-Climp63 were generated from GFP-Rtn4a and HA-Climp63, respectively, in pmCherry-C3 (a gift from Dong Li). mEmerald-Sec31A was PCR-amplified from a cDNA library in HEK293T cells and subcloned into pmEmerald-C1 (a gift from Dong Li). HA-ShhN was generated by cloning mouse Shh (1-198), followed by the 3xHA tag in pcDNA3.1 vector (Invitrogen). GFP-Sec16A was a gift from Xiaowei Chen. The RUSH expression constructs used in this study contained an ER hook, streptavidin-KDEL. The constructs encoding Str-KDEL_SBP-GFP-Vangl2, Str-KDEL_SBP-GFP-CTSZ, and Str-KDEL_SBP-GFP-α1-antitrypsin were generated by replacing the DNA fragment encoding EGFR within the plasmid Str-KDEL_SBP-EGFP-EGFR (a gift from Franck Perez) with a DNA fragment encoding mouse Vangl2, human CTSZ (24-304), and human α1-antitrypsin (25-419). Str-KDEL_VSVG-SBP-GFP was generated from Str-KDEL_SBP-EGFP-EGFR and pEGFP-VSVG (Addgene 11912). The plasmid encoding SBP-EGFP-ShhN was generated by replacing the DNA fragment encoding E-cadherin within the plasmid Str-

KDEL_SBP-EGFP-Ecadherin (Addgene 65286) with a DNA fragment encoding mouse ShhN (25-198).

To generate mEOS fusion proteins, calnexin, calreticulin, and the transmembrane domain of Sec61β (Sec61βTM) were PCR-amplified and subsequently cloned into pmEOS3.2-N1 (a gift from Pengyong Xu). To generate the mEOS-KDEL construct, the mEOS fragment was PCR-amplified with an N-terminal signal peptide of BiP and C-terminal ER retention signal (Lys-Asp-Glu-Leu, KDEL) and subcloned into pcDNA4/TO.

All plasmid inserts were confirmed by sequencing.

Genome editing using CRISPR/Cas9

ATL2 and ATL3 double knockout COS-7 cells were constructed as described previously (8). CRISPR targeting oligonucleotides designed for ATL2 (5′-GACGAGATCTTAACATAGTAGTGG-3′) and ATL3 (5′-GTTTTCACTGTGGAGAAGCCAGG-3′) were inserted into the pX330-U6-Chimeric_BB-CBh-hSpCas9 vector (Addgene 42230). Cells were co-transfected with CRISPR constructs, together with the pLKO.1 puro vector (Addgene 8453), and selected with 1 μg/mL puromycin (Invitrogen) for 1 week. The cells were then sorted as single cells into a 96-well plate using a FACS Aria-II sorter (BD BioSciences). Knockout efficiency was assessed by Western blot analysis and sequencing.

Genomic DNA was extracted using DNA extraction buffer (200 mM Tris (pH 8.3), 500 mM KCl, 5 mM $MgCl_2$, and 0.1% gelatin in H_2O). Gene-specific PCR was carried out with Taq DNA polymerase (Thermo) and primers specific for ATL2 (forward: 5′-AGTCCGACCAGAAGCTTG-3′ and reverse: 5′-CTTGTTATACATGTATCTAAGC-3′) and ATL3 (forward: 5′-ATTGGTTGGGTGACCCAGAAG-3′ and reverse: 5′-ACACAGTACATGCTGAAGTTC-3′). PCR products were resolved on a 1.5% agarose/1×

TAE gel, cut, and purified using a DNA gel extraction kit (Thermo). The purified PCR products were cloned into a pMD18-T vector (Takara) and sequenced.

Generation of stable shRNA knockdown cell lines

Annealed oligonucleotides were cloned into pLKO.1 puro cloning vector using AgeI and EcoRI cloning sites. The shRNA coding sequences are as follows: human ATL2, 5′-CCGGCAGTCATGTTTGCTATGTATACTCGAGTATACATAGCAAACATGACTGTTTTTTG-3′; human ATL3, 5′-CCGGCATCAGGTATTCTGGTCAATACTCGAGTATTGACCAGAATACCTGATGTTTTTTG-3′; and monkey Climp63, 5′-CCGGGCAGGATTTGAAAGCCTTGAACTCGAGTTCAAGGCTTTCAAATCCTGCTTTTTG-3′. HeLa and COS-7 cells stably expressing shRNA oligonucleotides were generated by lentiviral infection as described previously (9). Knockdown efficiency was assessed by Western blot analysis.

Transient siRNA-mediated knockdown

Small interfering RNA (siRNA) targeting ATL2 and ATL3 (1), OGT (10, 11), and Cul3 (12) and non-specific siRNA control were purchased from RiboBio. The siRNA sequences used in this study were: siATL2, 5′-GGAGCUAUCCUUAUGAACAUUCAUA-3′; siATL3, 5′-GGUUAGAGAUUGGAGUUUCCCUUAU-3′; siOGT#1, 5'-GGAGCCUUGCAGUGUUAUA-3'; siOGT#2, 5'-GAUUAAGCCUGUUGAAGUC-3'; siCul3#1, 5'-GAAGGAAUGUUUAGGGAUA-3'; and siCul3#3, 5'-CAUUAUUUAUUGAUGAUAA-3'. Cells were transfected with siRNAs using Lipofectamine RNAiMAX (Invitrogen) according to the manufacturer's instructions. All

experiments were performed 72 h after the first siRNA transfection. Knockdown efficiency was tested by quantitative real-time PCR (qRT-PCR) or immunoblot.

RNA isolation, RT-PCR, TA-cloning, and qRT-PCR

Total RNA was isolated using TRIzol reagent (Thermo Fisher Scientific) according to the manufacturer's protocol.

Reverse transcription was carried out with 1 μg RNA and oligo dT using reverse transcriptase (Promega). cDNA reverse-transcribed from poly-A mRNA was used as a template for PCR. Gene-specific PCR was carried out with Taq DNA polymerase (Thermo) and primers specific for Sec31A (forward: 5′-TGAACTTTGAGGATGATTC-3′, reverse: 5′-GGACTCTGTTCAAAGCTGGAGGGTC-3′). PCR products were resolved on a 1.5% agarose/1 × TAE gel, cut, and purified with a DNA gel extraction kit (Thermo). The purified PCR products were cloned into a pMD18-T vector (Takara) and sequenced.

Gene-specific primers for qRT-PCR were designed by using NCBI Primer-Blast (OGT: 5′-TCCTGATTTGTACTGTGTTCGC-3′ and 5′-AAGCTACTGCAAAGTTCGGTT-3′; Cul3: 5′-TGTGGAGAACGTCTACAATTTGG-3′ and 5′-GCGCCTCTGTCTACGACTT-3′). GAPDH was amplified using primers 5′-CCAGGGCTGCTTTTAACTC-3′ and 5′-TGGAAGATGGTGATGGGATT-3′ as an internal control. qRT-PCR amplifications were performed with 40 cycles using PowerUp SYBR Green Master Mix (Applied Biosystems) on an ABI-7300 real-time PCR machine (Applied Biosystems).

Mammalian cell culture, transfection, and immunofluorescence

COS-7 cells (ATCC) were maintained in Dulbecco's modified Eagle's medium (DMEM, Corning) supplemented with 10% fetal bovine serum (FBS, Gibco) at 37 ℃ in 5% CO_2.

For immunofluorescence experiments, cells were seeded on coverslips and transfected using Lipofectamine 3000 (Invitrogen) based on the manufacturer's instructions. Twenty-four hours after transfection, the cells were fixed in 4% paraformaldehyde (PFA) followed by permeabilization with 0.1% Triton X-100 (Bio Basic, Inc.) for 10 minutes (except for co-staining with antibodies against Sec16A and β-COP). Staining using commercially available antibodies directed against Sec16A and β-COP was preceded by fixation and permeabilization using ice-cold methanol at -20 ℃ for 5 min. Cells were washed twice with PBS and blocked with 3% BSA for 1 h at room temperature before probing with primary antibodies for 1 h. Cells were washed in PBS, and then incubated with various fluorophore-conjugated secondary antibodies for an additional 1 h. Cells were then washed and mounted on slides using mounting medium (KPL). Confocal imaging was performed on a LSM700 microscope (Zeiss, Thornwood, NJ). Images were scanned using a 63 × 1.4 numerical aperture (NA) oil objective lens. The confocal settings used for image capture were held constant when samples were being compared.

The following antibodies were used: rabbit anti-Sec31A (Bethyl), mouse anti-Sec31A (BD Bioscience), rabbit anti-Sec16A (Bethyl), Alexa Fluor 488 dye (Molecular Probes, Thermo) conjugated anti-Sec16A (Abcam, conjugated according to the manufacturer's instructions), rabbit anti-Sec24C (Abcam), Alexa Fluor 555 dye (Molecular Probes, Thermo) conjugated anti-Sec24C (Abcam, conjugated according to the manufacturer's instructions), rabbit anti-calreticulin (Abcam), rat anti-HA (1:500; Sigma), rabbit anti-Myc (Sigma), and rabbit anti-β COP (Thermo). Goat Alexa Fluor–conjugated secondary antibodies were purchased from Thermo Fisher Scientific. Brightness and contrast were adjusted across the entire image using Adobe Photoshop (identical for control and experimental conditions in groups compared).

Super-resolution 3D-SIM imaging

3D-SIM images were acquired on the DeltaVision OMX V3 imaging system (Applied Precision, GE) with a 100 × 1.40 NA oil objective (Olympus UPlanSApo), solid-state multimode lasers (488 and 561 nm), and electron-multiplying charge-coupled device cameras (Evolve 512 × 512, Photometrics). Serial *Z*-stack sectioning was performed at 125-nm intervals in SIM mode. SIM image stacks were reconstructed using software 6.1.1 (Applied Precision) with the following settings: pixel size 39.5 nm, channel-specific optical transfer functions, Wiener filter constant 0.0010, discard negative intensities background, drift correction with respect to first angle, and custom K0 guess angles for camera.

ER content was assessed as intensity of green fluorescence (ER marker) per selected region. The fluorescence was calculated by ImageJ.

The 'Spots' function was used to quantify Sec31A- or Sec24C-labeled structures in Imaris 8.1.2 (Bitplane^{TM}). The spot size (puncta diameter) was set at 0.12 μm as determined by the direct measurement of images. Background subtraction was selected. Following automatic detection of puncta, the threshold was manually adjusted (usually within 5% among different samples) with visual inspection to ensure that all puncta were identified, minimal background staining or noise was recognized, and that individual puncta were not double-counted. In all, the resultant count was recorded as the number of puncta. To ensure that user-introduced noise in threshold adjustment did not introduce significant bias, each image was quantified a second time, at a later time, without access to the prior quantification. If the difference exceeded 5%, the sample was discarded. GraphPad Prism6 was used to create the final bar charts.

Quantification of Pearson's colocalization coefficient

COS-7 cells were fixed and stained with the indicated antibodies. Stained cells were analyzed using a Zeiss LSM700 confocal microscope and processed by Zen software. The co-localization coefficient of Sec31A/Sec16A, Sec31A/Sec24C, and Sec24C/Sec16A was calculated by co-localization plugin Coloc2 in Fiji-ImageJ (13) in a 100 μm^2 region away from the nucleus after thresholding. The lower limit of intensity thresholds was defined by staining with secondary antibodies alone.

RUSH transport assay

COS-7 cells were grown on 15-mm glass coverslips in 12-well culture dishes for 24 h. The cells were then transfected with RUSH constructs using calcium phosphate (Promega) for 18 h according to the manufacturer's instructions. Release of the RUSH reporters was induced by the addition of biotin (Sigma) at a final concentration of 40 μM in the culture medium as described previously (14). The release was performed in the presence of 100 μg/mL cycloheximide (Merck-Millipore) to stop new protein synthesis. In parallel, a control sample without biotin was used for each condition to monitor the leakiness of the RUSH construct-encoded cargo protein before release (0 min). After the release, cells were fixed at the indicated time points. Images were acquired by Zeiss LSM700 confocal microscopy.

Fluorescence recovery after photobleaching (FRAP) assay

COS-7 cells were seeded and grown overnight in 15-mm Glass Bottom Cell Culture Dishes (NEST) in complete growth medium. After reporter protein transfection for 24 h, the cells were ready for FRAP. FRAP experiments were performed at 37 ℃ in 5% CO_2 on a Zeiss LSM880 confocal microscope with a 63 × 1.4 NA oil objective lens and a stage incubator system (PeCon GmbH, Erbach, Germany) and processed with Zen software. A 5-μm-diameter (2.5 μm in Fig. 4E) circular region of interest (ROI) was defined. Two pre-bleach

images were acquired, followed by bleaching with 100% laser power and 50 scanning iterations. Pre- and post-bleach images were acquired every 5 s (in Fig. 4E, images were acquired every 1 s). For cells transfected with RUSH constructs, cells were imaged immediately after biotin addition. For quantitative analysis of FRAP experiments, fluorescence intensity values were exported from Zen software. FRAP recovery curves were obtained in OriginPro8. Mobile fractions and t1/2 values were determined using a double exponential curve fit. The final data analysis was performed using GraphPad Prism6.

Fluorescence loss after photoswitching (FLAP) assay

COS-7 cells were cultured and transfected as described above. Fluorescence images were obtained using an Olympus FV1200 laser scanning confocal microscope equipped with an IX81 microscope with a 60 × 1.40 NA oil objective (Olympus, Tokyo, Japan) and a stage incubator system (Tokai Hit, Fujinomiya, Japan). The stage was heated at 37 ℃. Reporters were photon-converted using the SIM scanner in tornado scan mode with the 405-nm laser set at 5% transmission for 80 scanning iterations, and imaged using the 488 nm and 546 nm lasers. To reduce inter-cell variability, reporters were photo-converted in a 10 μm × 15 μm ROI next to the nucleus with a similar density as the ER in each cell. The fluorescence intensities within the ROI and cell-free area (background) were measured using the Olympus FV10-ASW software. After background subtraction, the red fluorescence intensity of the bleached region over time was normalized with the average fluorescence intensity of two frames before photobleaching and plotted as a function of time. Image analysis was performed in FV10-ASW2.0 and OriginPro8. The red fluorescence intensity was measured in each ROI. FLAP curves were obtained in OriginPro8.

Vesicular release assay

The vesicular release assay was described previously (4). One day after transfection, COS-7 cells grown in one 10-cm dish to approximately 70% confluence were permeabilized in 3 mL ice cold KOAc buffer (110 mM potassium acetate, 20 mM Hepes, pH 7.2, 2 mM magnesium acetate) containing 40 μg/mL digitonin on ice for 5 min, and the semi-intact cells were sedimented by centrifugation at 300 *g* for 3 min at 4 ℃. The cell pellets were resuspended in 1 mL high salt KOAc buffer (1M potassium acetate, 20 mM Hepes, pH 7.2, 2 mM magnesium acetate) and incubated on ice for 5 min. After incubation, the permeabilized cells were washed twice with 1 mL KOAc buffer and resuspended in 100 μL KOAc buffer. For experiments with the purified cytATL1, transfected cells were semi-permeabilized with 25 μg/mL digitonin on ice for 10 min, and indicated amounts of His-cytATL wt or R217Q in KOAc buffer were added at 30 ℃ along with other reagents. The budding assay was performed by incubating semi-intact cells (~0.02 OD/reaction) with 2 mg/mL rat liver cytosol in a 100 μL reaction mixture containing 200 μM GTP and an ATP regeneration system (40 mM creatine phosphate, 0.2 mg/mL creatine phosphokinase, and 1 mM ATP) in the presence or absence of 0.5 μg of dominant-negative Sar1A (H79G) mutant protein. After incubation at 30 ℃ for 1 h, the reaction mixture was centrifuged at 14000 *g* to remove cell debris and large membranes. The medium speed supernatant was then centrifuged at 100,000 *g* to sediment small vesicles. The pellet fraction was then resuspended in 100 μL of 35% OptiPrep and overlaid with 700 μL of 30% OptiPrep and 30 μL of KOAc buffer. The samples were centrifuged at 55,000 rpm in a TLS55 rotor in a Beckman ultracentrifuge for 90 min at 4 ℃. After centrifugation, the top fraction was collected and analyzed by Western blot.

Protein isolation and Western blot

Proteins were extracted with RIPA buffer (150 mM NaCl, 1% NP-40, 0.5% deoxycholic acid, 0.1% SDS, and 50 mM Tris, pH 8.0) containing protease and phosphatase inhibitors and analyzed by SDS-PAGE and Western blot. Proteins were detected using the following specific antibodies: rabbit anti-Sec31A (Bethyl), rabbit anti-ATL2 (Abcam), rabbit anti-ATL3 (Abcam), rabbit anti-Myc (Sigma), rabbit anti-Sec13 (Proteintech), rabbit anti-Sar1A (Proteintech), rabbit anti-Sec24C (Cell Signaling Technology), rabbit anti-Sec23A (Cell Signaling Technology), rabbit anti-HA (Cell Signaling Technology), mouse anti-Myc (Cell Signaling Technology), mouse anti-PDI (Enzo), mouse anti-actin (Abcam), mouse anti-tubulin (Thermo), rabbit anti-GAPDH (Sigma) rabbit anti-GFP (Proteintech), rabbit anti-HSP90 (Proteintech), rabbit anti-calreticulin (Abcam). Antibodies against Sec22B, ERGIC53 and Ribophorin I are gifts from Randy Schekman's lab (UC Berkeley). All commercial antibodies were validated by the manufacturer.

Immunoprecipitation

COS-7 cells were transfected with plasmids encoding GFP or GFP-Sec16A for 24 h. Cells were washed with PBS and lysed in 50 mM Tris-HCl (pH 7.5), 150 mM NaCl, 1% Triton X-100, and protease inhibitor. Lysates were spun at 12,000 rpm for 10 min and the supernatant incubated with anti-GFP agarose beads (Sigma) for 1 h at 4 ℃. The beads were collected by simple centrifugation and washed twice with lysis buffer. The cell lysates and immunoprecipitated proteins were subjected to SDS-PAGE followed by Western blot.

Sample preparation for mass spectrometry analysis

Vesicle samples were collected from the in vitro vesicle formation assay and resuspended with 0.1% Rapigest in 20 µl 50 mM TEAB resuspension buffer by vortexing for approximately 5 min. Urea (8 M, 20 µL) was added to the protein samples and vortexed

thoroughly for ~5 min. TCEP (10 mM) was added to the samples and incubated at 37 ℃ for 1 h and alkylated with 20 mM IAA at RT in the dark for 30 min. For trypsin digestion, the samples were diluted with 50 mM TEAB buffer three times and incubated with trypsin at 37 ℃ overnight. The next day, 10% TFA was added to a final concentration of 0.5% for 30 min at 37 ℃ to breakdown Rapigest at pH 2.5-3 and deactivate trypsin. Degraded Rapigest was removed after centrifugation at 13,000 rpm for 10 min. Samples were dried in a Speedvac concentrator overnight. The sample was then reconstituted in 0.1% TFA for desalting using the Pierce C18 spin column. The columns were activated with 50% ACN in 0.1% TFA, and then equilibrated by 0.1% TFA. The sample solution was added on top of the resin bed and washed with 0.1% TFA equilibration solution. Samples were collected by elution buffer with 50% and 75% ACN in 0.1% TFA. Finally, the samples were dried in a Speedvac concentrator and stored at -20 ℃ for further MS analysis.

Mass spectrometry and data analysis

An Acclaim PepMap RSLC C18 capillary column (75 μm × 25 cm; 2 μm particles, 100 Å) (Thermo fisher Scientific, San Jose, CA) was used for LC separation, and gradient elution was performed using an Ultimate 3000 nanoLC system (Thermo fisher Scientific, San Jose, CA) with a flow rate of 300 nl/min. Mobile phase A was 0.1% formic acid in water and mobile phase B 0.1% formic acid in acetonitrile. The analytical gradient lasted for 90 min (after 10 min of equilibration with 3% B, the composition of solvent B increased from 3% to 7% in 2 min, from 7% to 20% in 50 min, and from 20% to 30% in 2 min, followed by a washing and equilibration step when solvent B increased to 80% in 1 min and was held for 8 min, and then returned to 3% B in 0.1 min and was held for 17 min). An Orbitrap Fusion Lumos mass spectrometer (Thermo Fisher Scientific, San Jose, CA) operating in positive ion mode

was used for analysis. The ESI spray voltage was set at 2300 KV and the ion transfer tube temperature at 300 ℃. High resolution Orbitrap was used for both MS and MS/MS scans, with resolution at 60K and 15K, respectively. Data-dependent acquisition (DDA) mode was performed with a cycle time of 3 s. The mass range of the full MS scan defines *m/z* 400 to 1600, and in MS/MS starts from *m/z* 110. The collision energy was set at 30%. Three biological repeats of each sample were performed.

Proteome Discoverer 2.2.0.388 was used for protein identification and quantification with the following settings: (1) fixed modification: cysteine carbamidomethylation (+57.021 Da); (2) dynamic modification: methionine oxidation (+15.995 Da) and acetylation (+42.011) at the N terminus of the protein; (3) trypsin was used for digestion with one missed cleavage allowed; (4) peptide ion tolerance: 10 ppm; (5) fragment ion tolerance: 20 ppm; (6) the protein sequence of *Chlorocebus sabaeus* was downloaded from Uniprot (updated 11-2018) for database searching and identification with a false discovery rate (FDR) <0.01 considered reliable; (7) label-free quantification (LFQ) was performed using the ratio of the intensity of precursor ions. Only unique and razor peptides of proteins were selected for protein relative quantification. (8) Normalization to the protein median of each sample was used to correct experimental bias based on the total peptide amount. (9) Student's t-test was used to compare the significant changes between samples.

Statistical analysis

Averages and SEMs from at least three independent experiments are shown in figures when applicable. Sample sizes were chosen without performing statistical tests, but are based on studies with similar experimental designs and on the known variability of the assay. The data are presented as the mean ± SEM. Significance was determined by a Student's t-test. All P

values <0.05 were considered significant. In figures, $*p < 0.05$, $**p \leq 0.01$, $***p \leq 0.001$.

Calculations were performed using GraphPad Prism6 software.

SI REFERENCES

1. Rismanchi N, Soderblom C, Stadler J, Zhu PP, & Blackstone C (2008) Atlastin GTPases are required for Golgi apparatus and ER morphogenesis. *Hum Mol Genet* 17(11):1591-1604.
2. Wu FY, Hu XY, Bian X, Liu XQ, & Hu JJ (2015) Comparison of human and Drosophila atlastin GTPases. *Protein Cell* 6(2):139-146.
3. Bian X*, et al.* (2011) Structures of the atlastin GTPase provide insight into homotypic fusion of endoplasmic reticulum membranes. *P Natl Acad Sci USA* 108(10):3976-3981.
4. Merte J*, et al.* (2010) Sec24b selectively sorts Vangl2 to regulate planar cell polarity during neural tube closure. *Nat Cell Biol* 12(1):41-U94.
5. Ma TJ*, et al.* (2018) A mechanism for differential sorting of the planar cell polarity proteins Frizzled6 and Vangl2 at the trans-Golgi network. *J Biol Chem* 293(22):8410-8427.
6. Shibata Y, Voeltz GK, & Rapoport TA (2006) Rough sheets and smooth tubules. *Cell* 126(3):435-439.
7. Shibata Y*, et al.* (2008) The reticulon and DP1/Yop1p proteins form immobile oligomers in the tubular endoplasmic reticulum. *J Biol Chem* 283(27):18892-18904.
8. Sun S*, et al.* (2016) Identification of endoplasmic reticulum-shaping proteins in Plasmodium parasites. *Protein Cell* 7(8):615-620.
9. Zhu Y*, et al.* (2018) Sec61beta facilitates the maintenance of endoplasmic reticulum homeostasis by associating microtubules. *Protein Cell* 9(7):616-628.
10. Cho HJ & Mook-Jung I (2018) O-GlcNAcylation regulates endoplasmic reticulum exit sites through Sec31A modification in conventional secretory pathway. *Faseb J* 32(9):4641-4657.
11. Guo B*, et al.* (2014) O-GlcNAc-modification of SNAP-29 regulates autophagosome maturation. *Nat Cell Biol* 16(12):1215-U1202.
12. Jin LY*, et al.* (2012) Ubiquitin-dependent regulation of COPII coat size and function. *Nature* 482(7386):495-U213.
13. Schindelin J*, et al.* (2012) Fiji: an open-source platform for biological-image analysis. *Nat Methods* 9(7):676-682.
14. Boncompain G*, et al.* (2012) Synchronization of secretory protein traffic in populations of cells. *Nat Methods* 9(5):493-U120.

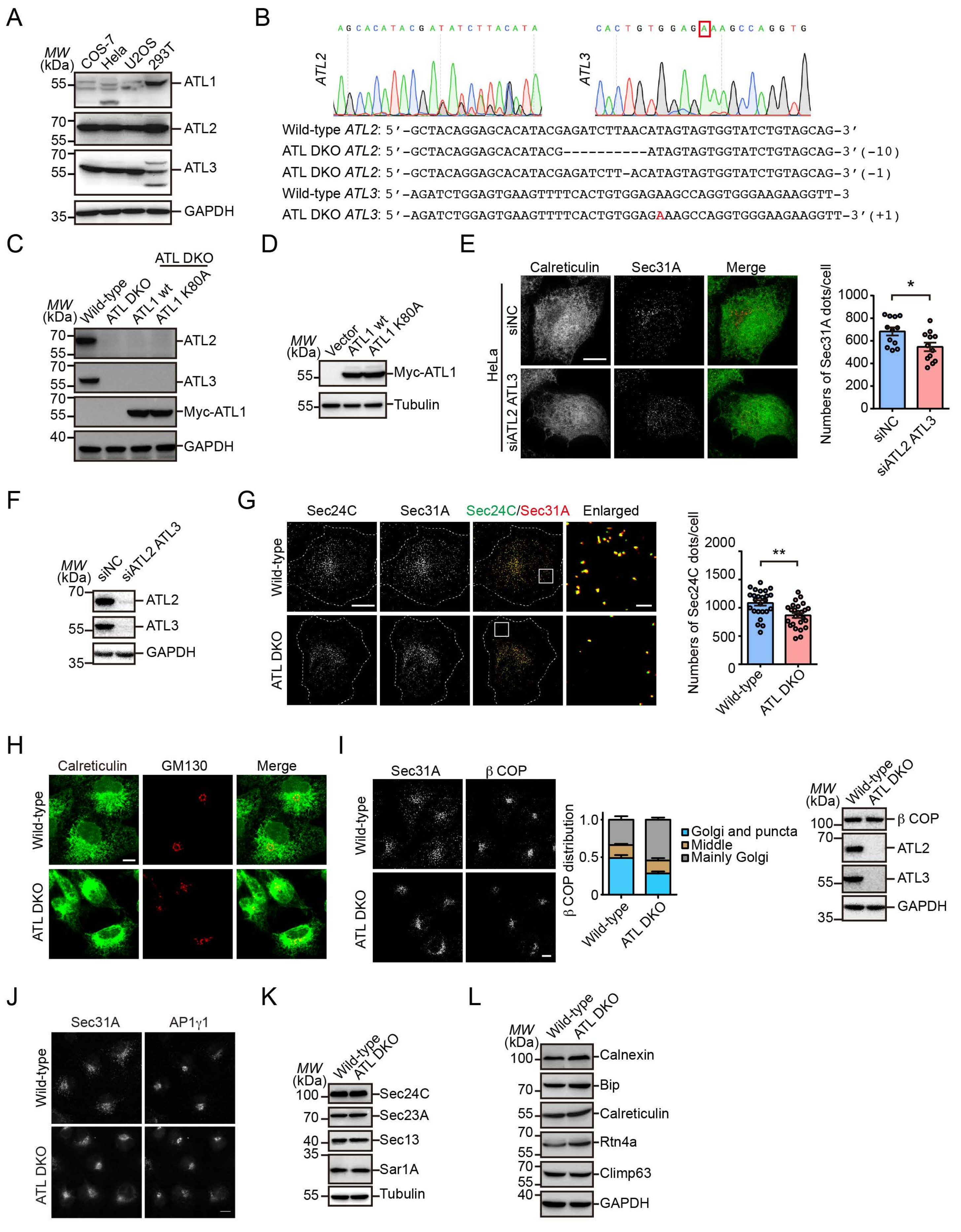

Figure S1. ATL deletion specifically affects COPII formation.
(A) Immunoblotting of cell lysates from COS-7, HeLa, U2OS, and 293T. Blots were probed to detect ATL1, ATL2, ATL3, or GAPDH as indicated. (B) Sequencing results for ATL2 and ATL3 in ATL DKO COS-7 cells. (C, D) Immunoblotting of cell lysates in Figure 1A and 1C. Blots were probed to detect ATL2, ATL3, Myc, or GAPDH. (E) HeLa cells were transfected with control siRNA (siNC) or ATL siRNAs (siATL2&3) for 48 h. Cells were fixed, stained using anti-calreticulin and anti-Sec31A antibodies, and visualized by SIM. Individual and merged images are shown. Scale bar: 5 μm. (*Right*) Quantification of Sec31A puncta (n =12 for each group). *p < 0.05 by two-tailed Student's *t*-test. (F) Immunoblotting of cell lysates in (E). Blots were probed to detect ATL2, ATL3, or GAPDH as indicated. (G) Wild-type COS-7 and ATL DKO COS-7 cells were fixed, stained using anti-Sec24C and anti-Sec31A antibodies, and visualized by SIM. Individual and merged images are shown. Scale bar: 5 μm. *Right*, quantification of Sec24C puncta (n = 20 for each group). *p < 0.05 by two-tailed Student's *t*-test. (H) Wild-type and ATL DKO COS-7 cells were fixed and immunostained using anti-calreticulin and anti-GM130 antibodies. Representative confocal images are shown. Scale bar: 10 μm. (I) (*Left*) Wild-type and ATL DKO COS-7 cells were fixed and immunostained using anti-β COP and anti-Sec31A antibodies. Representative confocal images are shown. Scale bar: 10 μ m. (*Middle*) Quantification of the distribution of β COP. (*Right*) Immunoblotting to detect β COP, ATL2, ATL3, or GAPDH as indicated. (J) Wild-type and ATL DKO COS-7 cells were fixed and immunostained using AP1γ1 and anti-Sec31A antibodies. Representative fluorescent images are shown. Scale bar: 10 μm. (K) Immunoblotting of COS-7 cell lysates from wild-type and ATL DKO COS-7 cells. Blots were probed to detect Sec13, Sec24C, Sec23A, Sar1A, or tubulin as indicated. (L) Immunoblotting of COS-7 cell lysates from wild-type and ATL DKO COS-7 cells. Blots were probed to detect calnexin, Bip, calreticulin, Climp63, Rtn4a, or GAPDH as indicated.

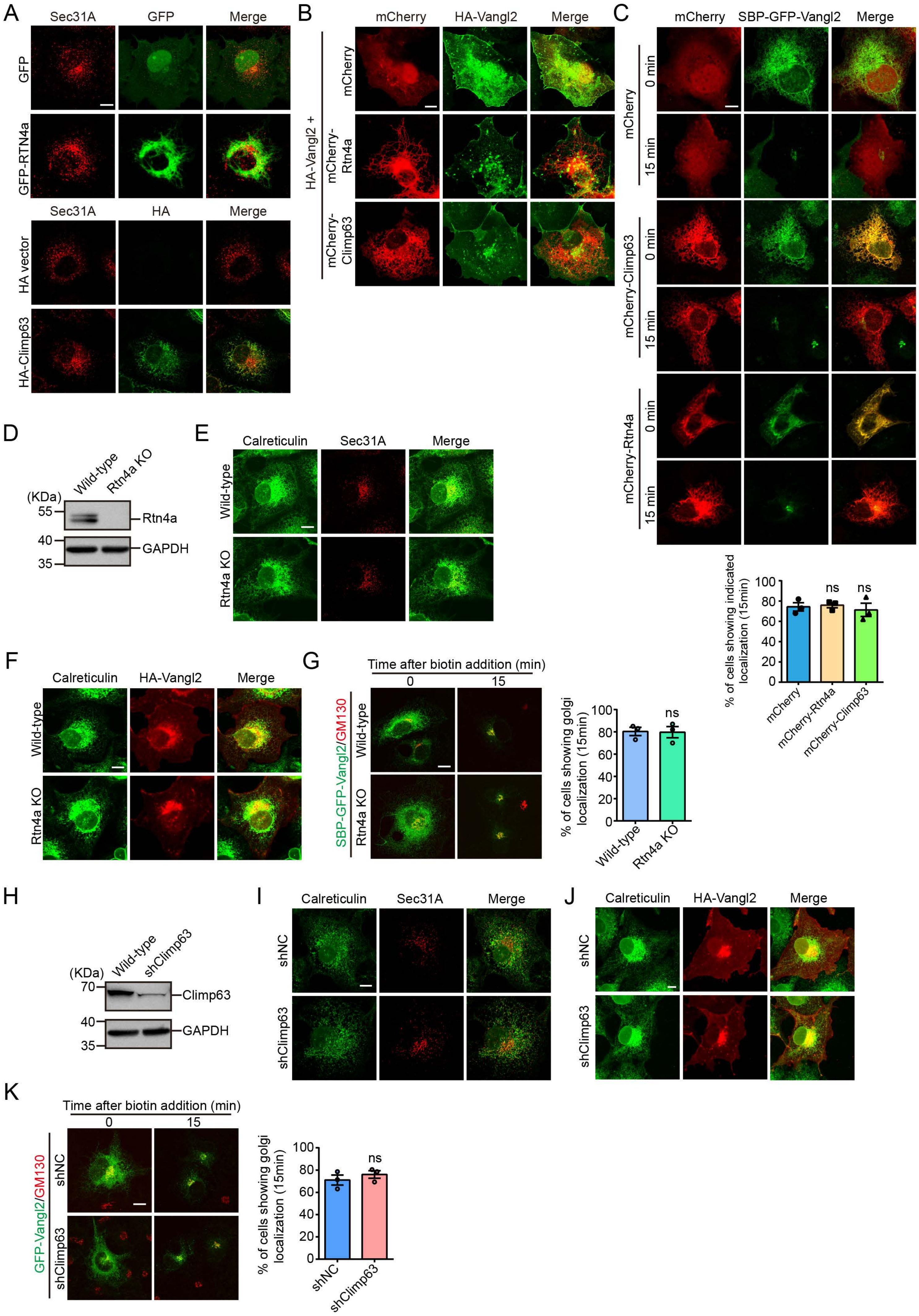

Figure S2. ATL deletion specifically affects ER export.
(A) Confocal images of COS-7 cells transfected with GFP-Rtn4a, HA-Climp63, or corresponding empty vectors for 24 h. Cells were fixed and stained with anti-Sec31A and anti-HA antibodies. (B) Representative confocal images of COS-7 cells co-transfected with HA-Vangl2 and mCherry vector, mCherry-Rtn4a, or mCherry-Climp63. (C) COS-7 cells were co-transfected with RUSH-Vangl2 and mCherry-Rtn4a or mCherry-Climp63. Trafficking of SBP-GFP-Vangl2 from the ER to the Golgi was analyzed using the RUSH system. The *bottom* graph is quantified data for SBP-GFP-Vangl2 localization at t = 15 min (n = 3, > 50 cells were counted for each experiment). (D) Immunoblotting of cell lysates from wild-type and Rtn4a KO COS-7 cells. Blots were probed to detect Rtn4a or GAPDH. (E) Wild-type and Rtn4a KO COS-7 cells were fixed and immunostained using anti-calreticulin and anti-Sec31A antibodies. Representative confocal images are shown. (F) Representative confocal images of wild-type and Rtn4a KO COS-7 cells transfected with HA-Vangl2. (G) Wild-type and Rtn4a KO COS-7 cells were transfected with RUSH-Vangl2. Trafficking of SBP-GFP-Vangl2 from the ER to the Golgi was analyzed using the RUSH system. The graph to the right is quantified data for SBP-GFP-Vangl2 localization at t = 15 min (n = 3, > 50 cells were counted for each experiment). (H) Immunoblotting of cell lysates from control (shNC) and shClimp63 COS-7 cells. Blots were probed to detect Climp63 or GAPDH. (I) As in (E), but with shNC and shClimp63 COS-7 cells. (J) As in (F), but with shNC and shClimp63 COS-7 cells. (K) As in (G), but with shNC and shClimp63 COS-7 cells. Scale bars: 10 μm. Quantified data represent mean ± SEM. ns, not significant by two-tailed Student's *t*-test.

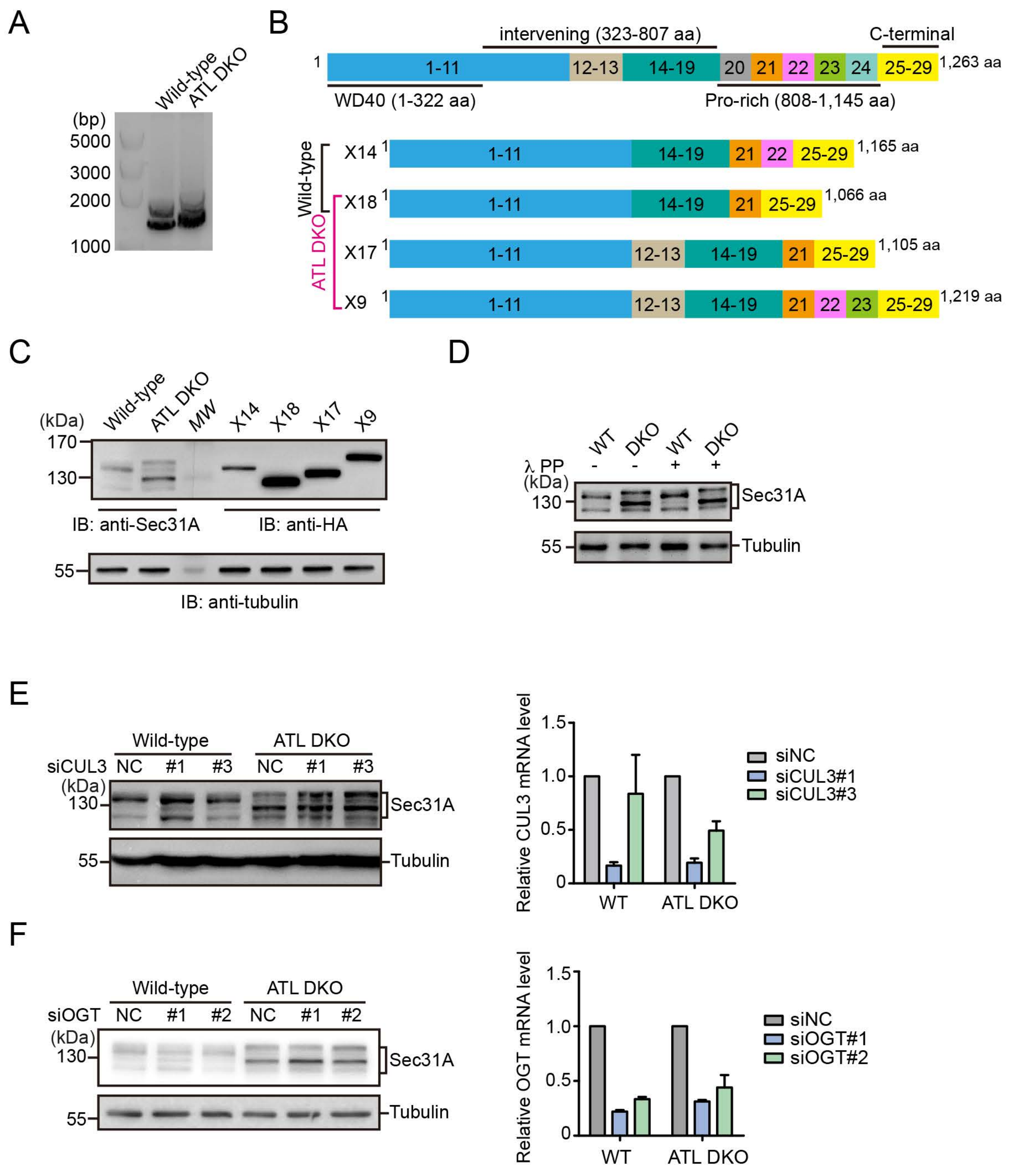

Figure S3. ATL deletion alters splicing of *Sec31A*.
(A) RT-PCR of Sec31A mRNA derived from wild-type and ATL DKO COS-7 cells were resolved on agarose gels. (B) (*Top*) Domain structure of the predicted longest Sec31A transcript in COS-7 cells. Numbers indicate exons, and exons are not to scale. (*Bottom*) Sequencing of TA-cloned PCR products (wild-type, 32 clones; ATL DKO, 37 clones) revealed five different Sec31A transcripts. X14 and X18 in wild-type COS-7 cells; X9, X17, and X18 in ATL DKO cells. (C) Immunoblotting of cell lysates from wild-type COS-7 cells, ATL DKO COS-7 cells, or COS-7 cells transfected with 2xHA tagged Sec31A X18, X17, X14, and X9. Blots were probed to detect Sec31A, HA, or tubulin as indicated. (D) Cell lysates from wild-type or ATL DKO COS-7 cells were incubated with Lambda Protein Phosphatase (λ PP, NEB) at 30°C for 30 min and analyzed by Western blot. Blots were probed to detect Sec31A and tubulin. (E) Wild-type and ATL DKO COS-7 cells were transfected with control siRNA (NC) or siRNAs targeting cullin3 (CUL3, #1 and #3) for 48 h. (*Left*) Cells were lysed and analyzed by Western blot. Blots were probed to detect Sec31A and tubulin. (*Right*) Relative CUL3 mRNA levels were determined by qPCR. Quantified data represent mean ± SEM. (F) As in (E), but with siRNAs targeting OGT transfection.

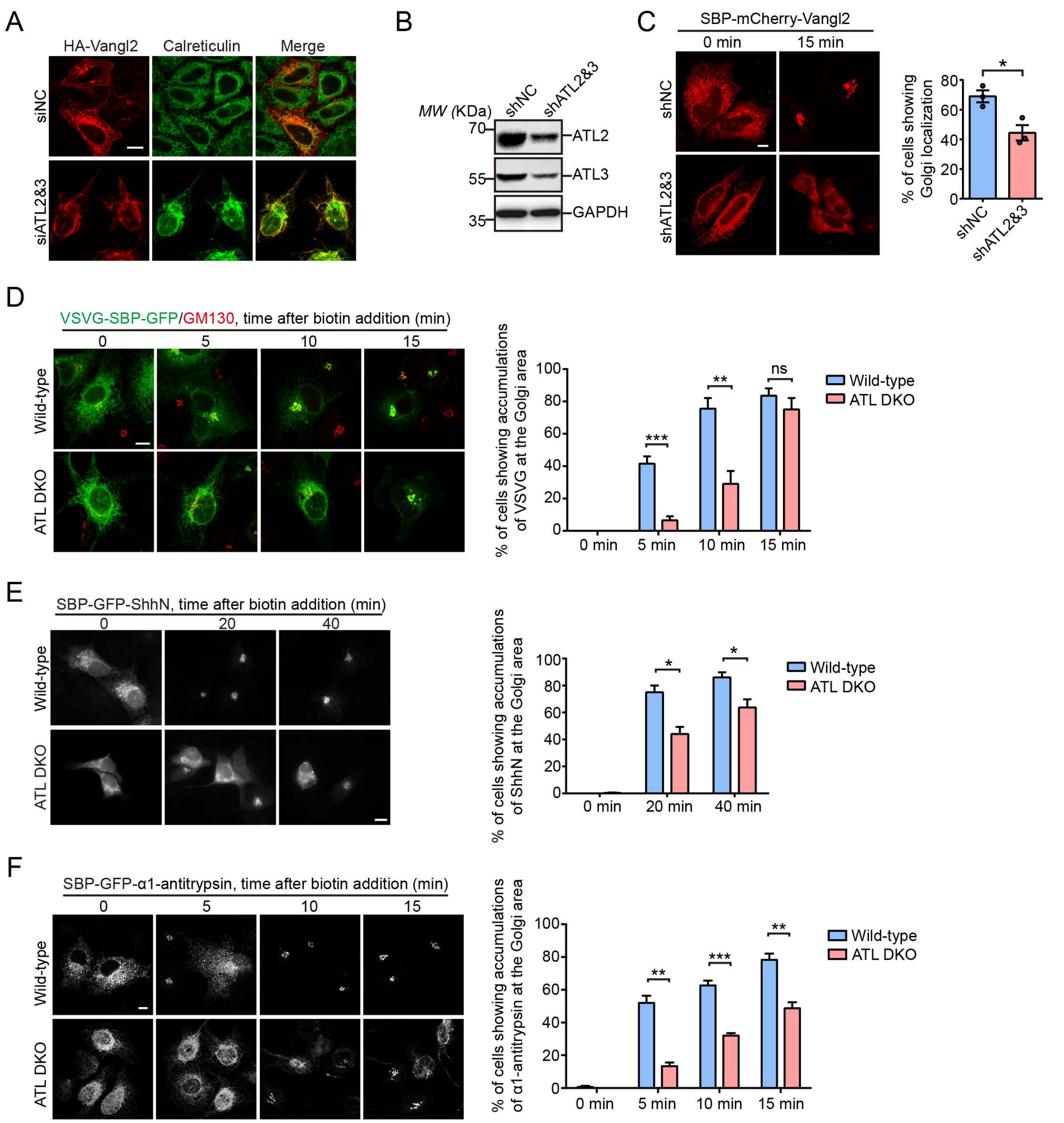

Figure S4. ATL deletion delays ER-to-Golgi trafficking.

(A) HeLa cells were co-transfected with HA-Vangl2 and control siRNA (siNC) or ATL siRNAs (siATL2&3) for 48 h. Cells were fixed and stained using anti-calreticulin and anti-HA antibodies. Representative confocal images are shown. (B) Immunoblotting of cell lysates from control (shNC) and shATL2&3 HeLa cells. Blots were probed to detect ATL2, ATL3, or GAPDH as indicated. (C) shNC and shATL2&3 HeLa cells were transfected with plasmids encoding RUSH-Vangl2. Day 1 after transfection, cells were incubated with biotin for the indicated period of time and the localization of SBP-GFP-Vangl2 analyzed by fluorescent microscopy. (*Right*) Quantification of the proportion of cells showing accumulation of SBP-GFP-Vangl2 at the Golgi area (n=3, > 50 cells were counted for each experiment, mean ± SEM). *p < 0.05 by two-tailed Student's *t*-test. ns, not significant. (D) Wild-type COS-7 cells and ATL DKO COS-7 cells were transfected with plasmids encoding RUSH-VSVG. Day 1 after transfection, cells were incubated with biotin for the indicated duration and the localization of VSVG-SBP-GFP analyzed by fluorescent microscopy. (*Right*) Quantification of the proportion of cells showing accumulation of VSVG-SBP-GFP at the Golgi area (n=3, > 50 cells were counted for each experiment, mean ± SEM). ***p < 0.001, **p < 0.05 by two-tailed Student's *t*-test. ns, not significant. (E) As in (D), but with RUSH-ShhN transfection. (*Right*) Quantification of the percentage of cells showing accumulation of SBP-GFP-ShhN at the Golgi area (n=3, > 50 cells were counted for each experiment, mean ± SEM). *p < 0.05 by two-tailed Student's *t*-test. (F) As in (D), but with RUSH-α1-antitrypsin transfection. (*Right*) Quantification of the proportion of cells showing accumulation of SBP-GFP-α1-antitrypsin at the Golgi area (n=3, > 50 cells were counted for each experiment, mean ± SEM). ***p < 0.001, **p < 0.05 by two-tailed Student's *t*-test. Scale bars: 10 μm.

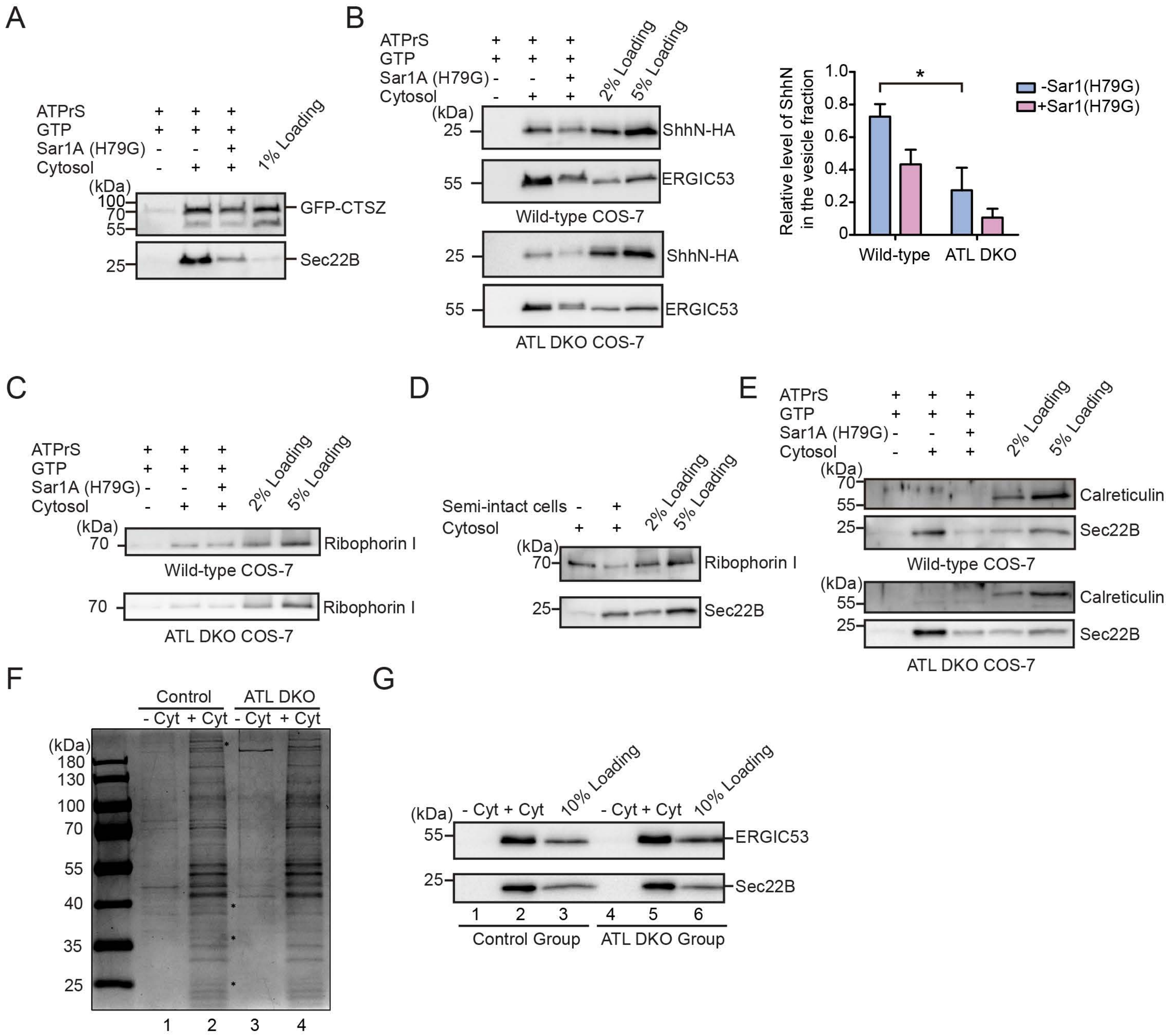

Figure S5. ATL deletion causes defects in packaging of ShhN into transport vesicles.
(A) Wild-type COS-7 cells were transfected with GFP-CTSZ. 24 h after transfection, the vesicle release reaction was performed using the indicated reagents. Vesicle fractions were then analyzed by immunoblotting using antibodies against GFP or Sec22B. (B) (*Left*) Wild-type COS-7 cells and ATL DKO COS-7 cells were transfected with plasmids encoding ShhN-HA. Day 1 after transfection, cells were incubated at 15°C for 2 h and the vesicle release reaction performed by incubating the digitonin-permeabilized cells with the indicated reagents. Vesicle fractions were then isolated and analyzed by immunoblotting. (*Right*) Quantification of the levels of ShhN-HA in the vesicle fraction normalized to the 2% loading (n=3, mean ± SEM). *$p < 0.05$ by two-tailed Student's *t*-test. (C) The vesicle release reaction was performed using wild-type COS-7 cells and ATL DKO COS-7 cells. Vesicle fractions were then isolated and analyzed by immunoblotting using antibodies probing ERGIC53 or Ribophorin I. (D) The vesicle formation assay was performed in the presence or absence of wild-type semi-intact COS-7 cells. Vesicle fractions were then isolated and analyzed by immunoblotting using antibodies probing Ribophorin I or Sec22B. (E) As in (C), but with antibodies probing calreticulin or Sec22B. (F, G) The vesicle release reaction was performed using wild-type COS-7 cells and ATL DKO COS-7 cells in the presence or absence of rat liver cytosol. Vesicle fractions were then isolated and analyzed by Coomassie Blue staining (F) or immunoblotted using the indicated antibodies (G). Asterisks (*) in (F) indicate protein bands with higher staining intensities in the Control group than the ATL DKO group.

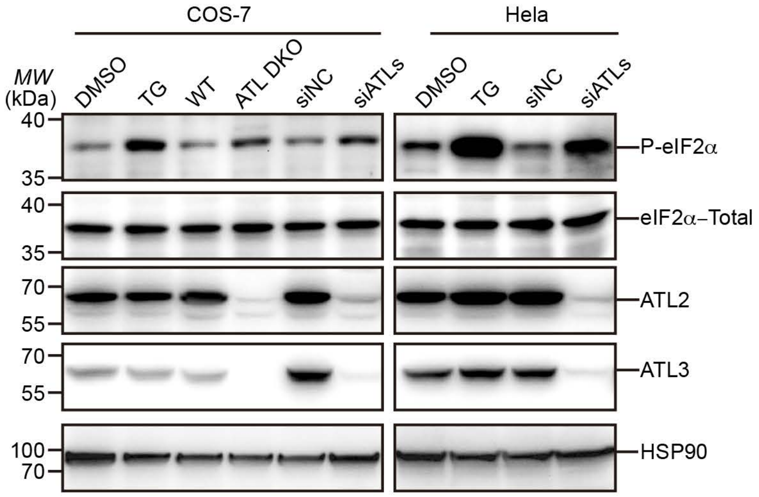

Figure S6. ATL deletion causes ER stress.
Lysates of indicated cells were analyzed by immunoblotting using various antibodies. For a positive control of ER stress, cells were treated with 2 μM thapsigargin (TG) for 2 h. Phosphorylation of eIF2α indicates levels of ER stress. HSP90 was the loading control.

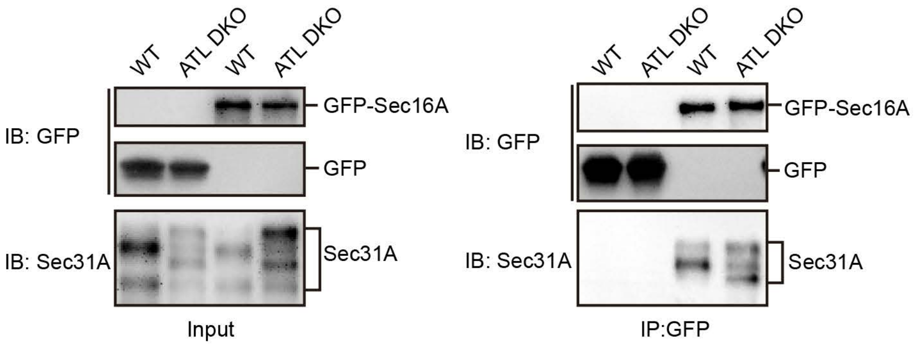

Figure S7. Sec16A interacts with Sec31A in ATL deleted cells.
Immunoblotting analysis of immunoprecipitation samples from GFP or GFP-Sec16A transfected wild-type and ATL DKO COS-7 cells. Input represents 5% of the total cell extract used for immunoprecipitation. Input samples (*left*) and anti-GFP immunoprecipitated samples (*right*) were blotted with anti-GFP and anti-Sec31A antibodies.

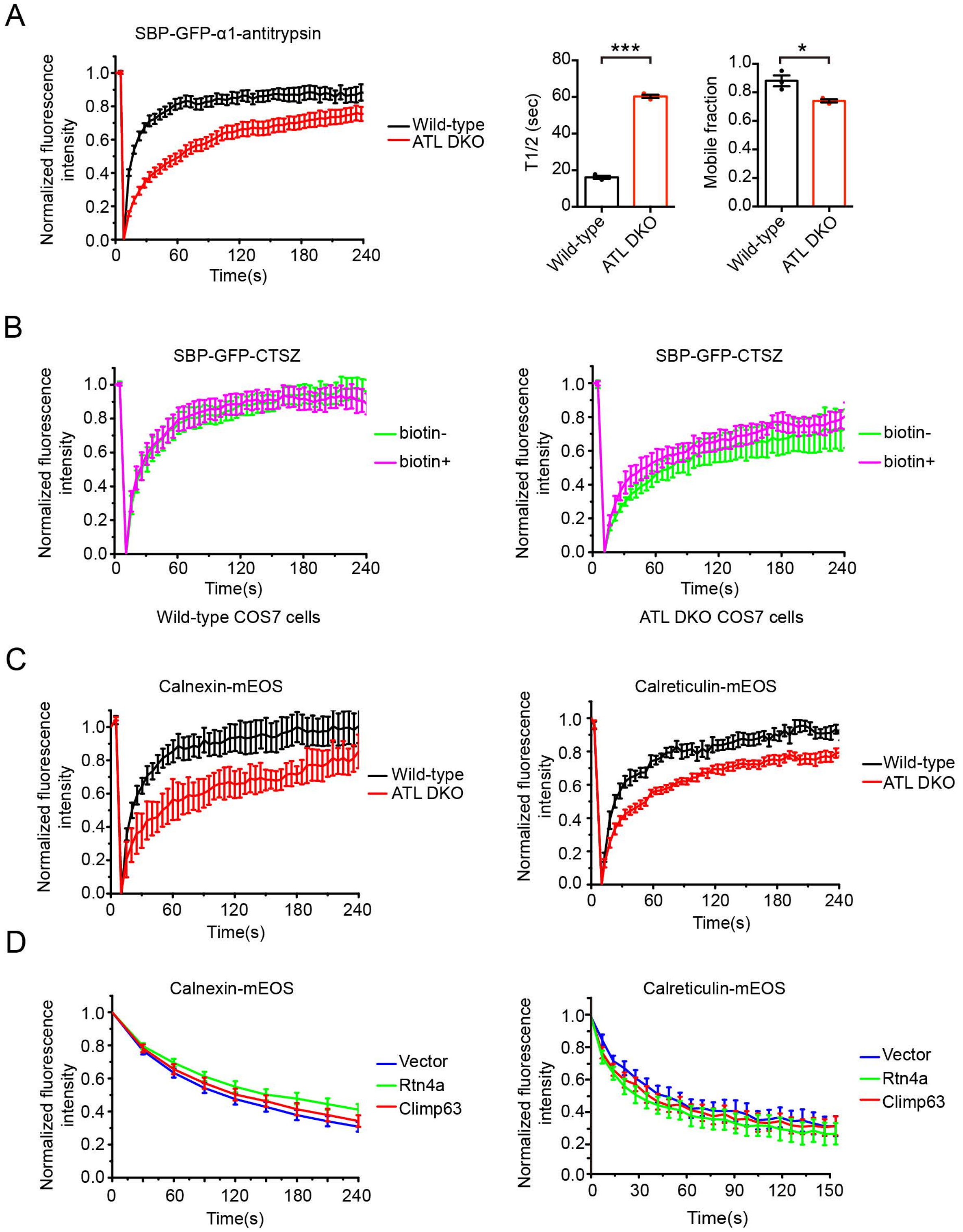

Figure S8. ATL regulates ER protein mobility.
(A) Wild-type and ATL DKO COS-7 cells were transfected with RUSH-GFP-α1-antitrypsin for 18 h. FRAP was performed immediately after biotin addition. Recovery curves (mean ± SEM), average recovery halftime (T1/2), and mobile fractions were derived from FRAP experiments. n = 3, 20 cells were counted for each group; bars represent mean ± SEM; *$p < 0.05$, ***$p < 0.001$ by two-tailed Student's *t*-test. (B) Wild-type and ATL DKO COS-7 cells were transfected with RUSH-CTSZ for 18 h and FRAP performed with or without biotin addition. (C) FRAP in the ER of wild-type and ATL DKO COS-7 cells transfected with calnexin-mEOS or calreticulin-mEOS plasmids. (D) FLAP in the ER of COS-7 cells co-transfected with reporter proteins and Rtn4a or Climp63. Calnexin-mEOS or calreticulin-mEOS were co-transfected with HA-Rtn4a, HA-Climp63, or empty vector. As in Figure 5, the fluorescence loss over time was measured in the photo-switched region. All curves show the mean fluorescence intensity ± SEM.

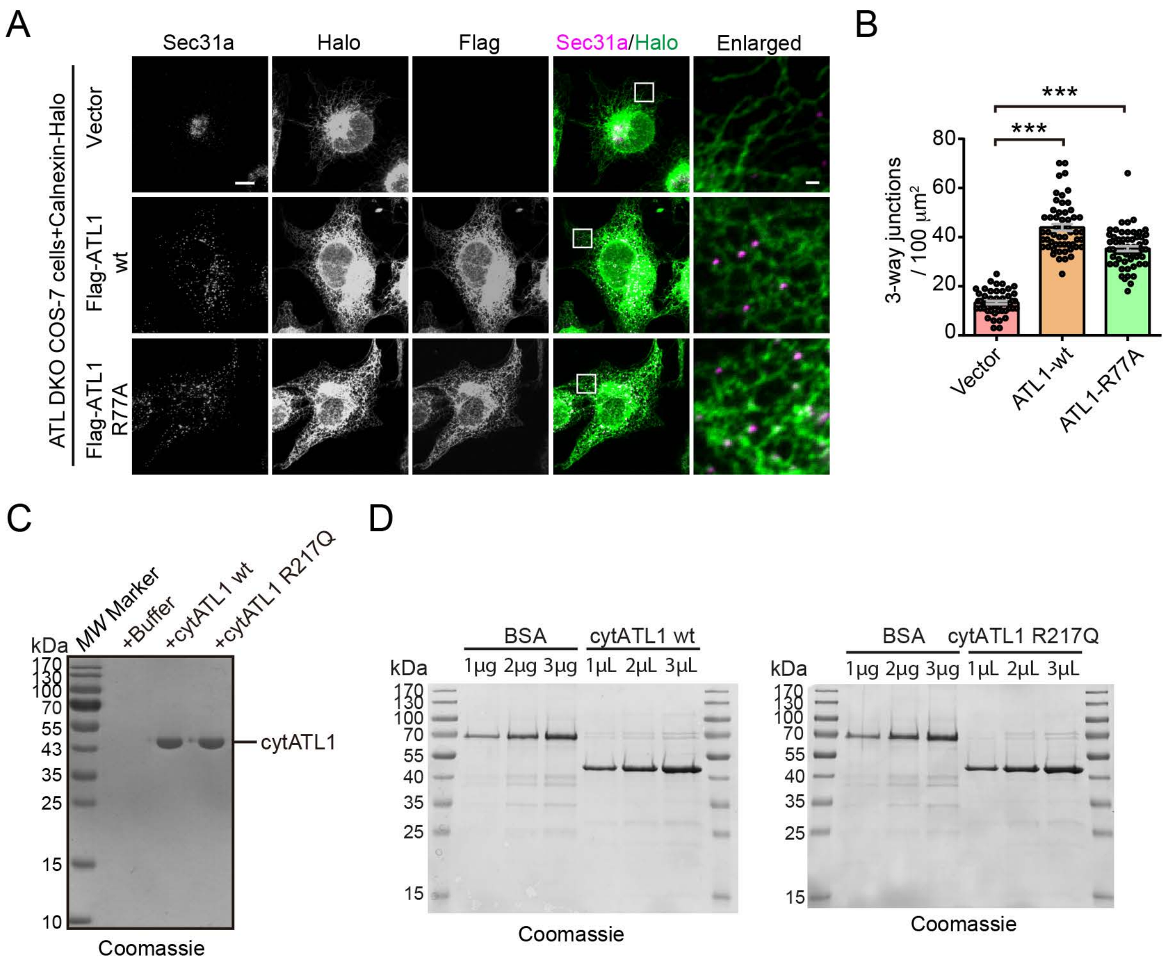

Figure S9. ATL-mediated membrane tethering.
(A) ATL DKO COS-7 cells were co-transfected with calnexin-Halo and vector, Flag-ATL1 wt, or Flag-ATL1 R77A. 24 h after transfection, cells were fixed and stained using anti-Flag and anti-Sec31A antibodies. Representative confocal images are shown. Scale bars: 5 μm or 1 μm for enlarged views. (B) Quantification of three-way junctions in a 100 μm^2 region away from the nucleus. For cells transfected with vector, n = 51; with ATL1 wt, n = 55; with ATL1 R77A, n = 60. Data are presented as mean ± SEM. ***$p < 0.001$ by two-tailed Student's *t*-test. (C, D) Purified His-cytATL1 wt or R217Q used in Fig. 6E and 6F were analyzed by Coomassie blue staining.
